# SRPS: Survival Reinforced Transfer Learning for Multicentric Proteomic Subtyping and Biomarker Discovery

**DOI:** 10.1093/gpbjnl/qzaf052

**Published:** 2025-06-10

**Authors:** Linhai Xie, Pei Jiang, Cheng Chang

**Affiliations:** State Key Laboratory of Medical Proteomics, Beijing Proteome Research Center, National Center for Protein Sciences (Beijing), Beijing Institute of Lifeomics, Beijing 102206, China; International Academy of Phronesis Medicine (Guangdong), Guangzhou 510320, China; State Key Laboratory of Medical Proteomics, Beijing Proteome Research Center, National Center for Protein Sciences (Beijing), Beijing Institute of Lifeomics, Beijing 102206, China; International Academy of Phronesis Medicine (Guangdong), Guangzhou 510320, China; State Key Laboratory of Medical Proteomics, Beijing Proteome Research Center, National Center for Protein Sciences (Beijing), Beijing Institute of Lifeomics, Beijing 102206, China

**Keywords:** Transfer learning, Subtyping, Proteomics, Biomarker discovery, Peptidyl-prolyl *cis-trans* isomerase C

## Abstract

Omics-based molecular subtyping in large-scale and multicentric cohort studies is a prerequisite for proteomics-driven precision medicine (PDPM). However, maintaining subtypes with robust molecular features and significant prognostic associations across different cohorts remains challenging due to biological heterogeneity and technical inconsistency. Herein, we propose a subtyping algorithm, named Survival Reinforced Patient Stratification (SRPS), to adapt known subtypes from a discovery cohort to another by simultaneously preserving the distinct prognosis and molecular characteristics of each subtype. SRPS was benchmarked on simulated and real-world datasets, demonstrating a 12% increase in classification accuracy and best prognostic discrimination. Moreover, based on the calculated subtype significance score, an “unpopular” protein, peptidyl-prolyl *cis*-*trans* isomerase C (PPIC), was identified as the top 1 remarkable protein for subtyping hepatocellular carcinoma (HCC) patients with the worst prognosis. Eventually, PPIC was experimentally validated as a pro-cancer protein in HCC, confirming our work as a demonstration of interpretable machine learning-guided biological discovery in PDPM research. SRPS is publicly available at https://github.com/PHOENIXcenter/SRPS and https://ngdc.cncb.ac.cn/biocode/tool/BT007770.

## Graphical abstract

**Figure:**
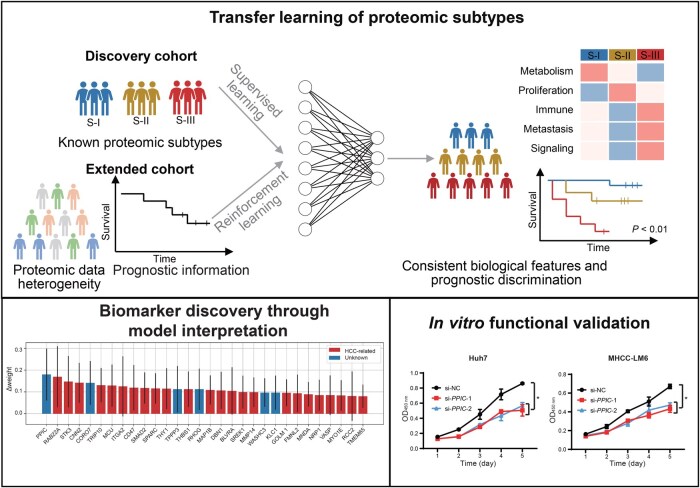

## Introduction

Proteomic subtyping has been broadly applied in oncology research on large cohorts, with an important goal of stratifying patients with diverse prognostic profiles according to the proteomic features [1–6]. Thorough analysis of differentially expressed proteins between subtypes is able to suggest the potential prognosis biomarkers and drug targets for clinical applications. Consequently, the research paradigm of proteomics-driven precision medicine (PDPM) was proposed [1]. However, to deliver the molecular stratification of patients from a scientific discovery to a clinical application, a fundamental step is to expand its validity to multiple independent clinical cohorts [7–10] to eliminate the sample bias in a single cohort with a relatively small sample size. Unfortunately, such an expansion of existing molecular subtypes on multiple cohorts is difficult due to unavoidable inter-cohort data heterogeneity that stems from both biological divergence and technical inconsistency. Hence, the problem we investigate in this study is how to transfer prognosis-discriminative molecular subtypes from a source cohort to a target cohort. We refer to this process as cohort adaptation for molecular subtyping in large-scale and multicentric cohorts.

Commonly applied supervised classification approaches [11,12] lack generalization ability between data with biased distributions. Correspondingly, batch effect removal algorithms [13,14] have been proposed to integrate data partially affected by batch effects, albeit with the risk of over-correction that may lead to unreliable downstream analyses [15]. Another alternative is domain adaptation in transfer learning [16–18]. It can search for data representations that are invariant between cohorts while maintaining distinctiveness for each subtype in the source cohort. Nevertheless, its drawback in our scenario is that it fails to effectively preserve the prognostic discrimination of subtypes from the source cohort to the target cohort.

**Figure 1:**
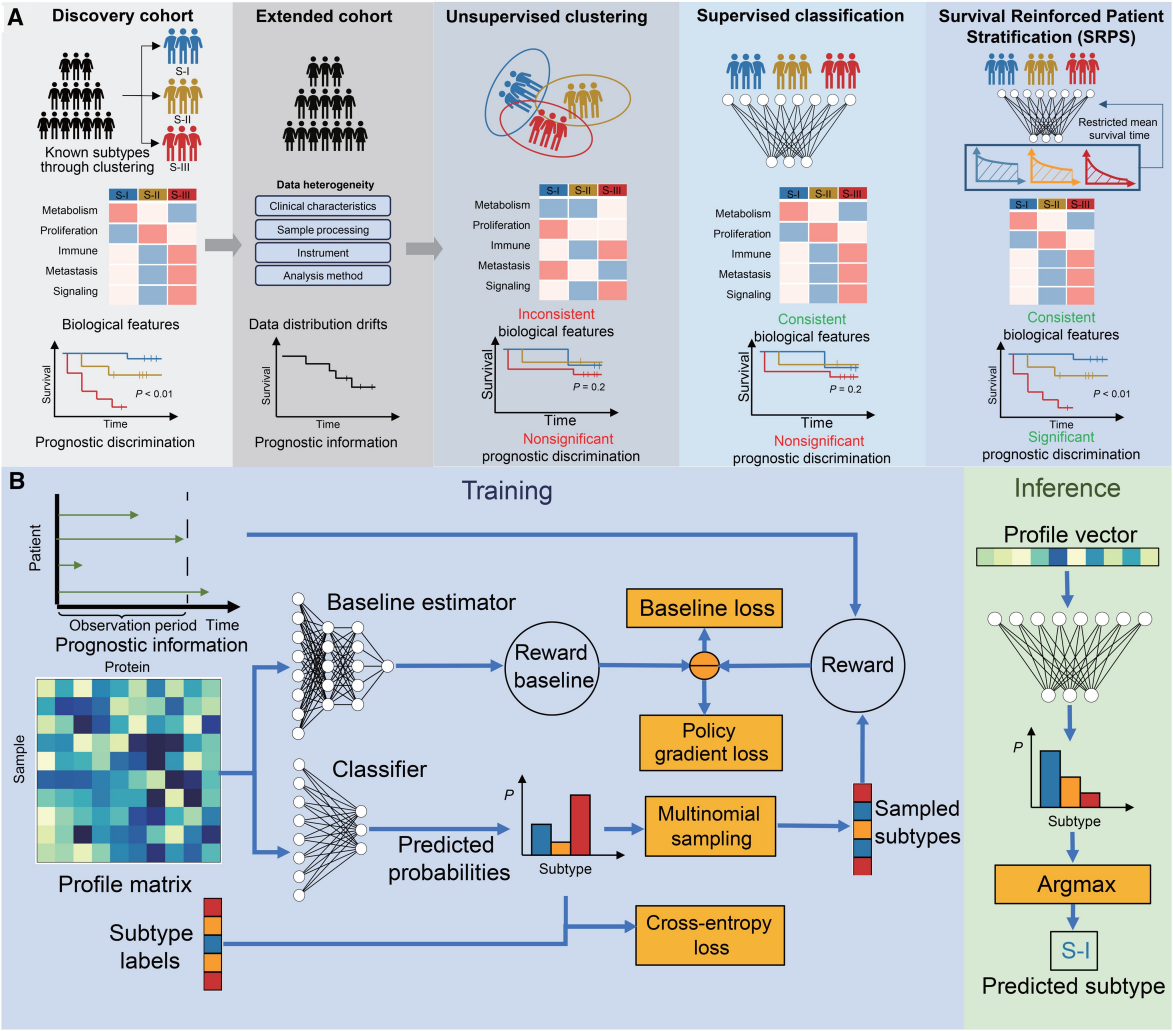
The innovation and learning strategy of SRPS **A**. Challenges of transferring known subtypes to new cohorts. When transferring subtypes with known biological features and prognostic discrimination from a discovery cohort to an extended cohort with batch effects, existing methods face significant limitations. Unsupervised clustering may not recover consistent biological features of known subtypes, while supervised classification often fails to preserve prognostic discrimination. SRPS addresses these challenges by simultaneously learning from the subtype labels of the discovery cohort and the survival data of the extended cohort. This dual-learning approach enables SRPS to preserve both the biological features and the prognostic discrimination of the subtypes. **B**. Training and inference workflow of SRPS. During training, SRPS optimizes a classifier to predict patient subtypes based on molecular profiles using two complementary learning paradigms. In the discovery cohort, supervised learning minimizes prediction errors relative to known subtype labels. In the extended cohort, on-policy reinforcement learning maximizes prognostic discrimination between subtypes by optimizing a policy gradient. ⊖ represents element-wise subtraction. In the inference phase, SRPS assigns a predicted subtype to each patient using the trained classifier, as exemplified by the predicted liver cancer subtype (S-I). SRPS, Survival Reinforced Patient Stratification.

As illustrated in **Figure 1**A, we propose a novel algorithm named Survival Reinforced Patient Stratification (SRPS) to adapt prognostically discriminative patient stratification from a source cohort to a target cohort. SRPS optimizes a classifier to predict the subtype of a patient exclusively based on the molecular profile through two learning paradigms. In the source cohort, it learns the biological features of known subtypes through supervised learning, while in the target cohort, it learns to maximize the prognostic discrimination between subtypes through reinforcement learning. Hence, SRPS can preserve both the proteomic features and prognostic significance of each subtype when transferring the subtypes between cohorts.

The performance of SRPS was benchmarked against five baseline methods on both simulated and real-world datasets (Table S1). On simulated datasets, SRPS outperformed all baseline methods on the target cohort with significant batch effects, achieving average improvements of 12% in classification accuracy and nearly 100% in log-rank score. On real-world datasets, SRPS demonstrated comparable accuracy on the source cohort and maintained single-sample gene set enrichment analysis (ssGSEA) similarity between the source and target cohorts, while achieving the highest survival discrimination in both overall survival (OS) and recurrence-free survival (RFS) clinical outcomes.

Furthermore, model interpretability is essential for biological and clinical applications of machine learning [19,20], and several strategies [21–23] have been proposed to interpret complex models. For this purpose, we designed the network architecture of the classifier with only a single dense layer, which enabled the straightforward translation of model parameters into a subtype significance score. By applying this scoring function to models that transferred subtypes between two hepatocellular carcinoma (HCC) cohorts, we observed insightful reasons for the enhanced prognostic discrimination of the classifier. It allocated higher weights on proteins which are originally prognostic discriminators. More importantly, the scoring function indicated peptidyl-prolyl *cis*-*trans* isomerase C (PPIC) as the most significant protein when identifying a group of HCC patients with the worst prognoses.PPIC successfully stratified patients into two groups with significantly different prognoses in two HCC cohorts for OS and RFS, and for the first time, its role in promoting proliferation, migration, and colony formation of HCC cells was experimentally validated. These results suggest a potential clinical application of PPIC as a prognostic biomarker.

## Method

### Survival Reinforced Patient Stratification

As illustrated in Figure 1B, the training framework of SRPS consists of a subtype classifier and a reward baseline estimator. The classifier predicts the subtyping probabilities of patients given the proteomic profile matrix. For data with subtype labels in the source cohort, a cross-entropy loss is calculated using both the predicted probabilities and the provided ground truth. By minimizing this objective, the classifier learns the principal proteomic pattern of each subtype, enabling it to stratify patients accordingly.

More specifically, given $x_{i}\in R^{m}$ to represent the m-dimensional proteomic profile vector of sample i∈I where I is the patient set, a prediction vector is calculated as follows:


(1) $$p_{i}=f_{\text{sm}}(f_{c}(x_{i})|\Theta )$$

Each $p_{i}^{\left(s\right)}$ in $p_{i}$ represents the probability of patient i belonging to subtype s ∈ {1, 2, …, k}, supposing k different subtypes in total. $f_{\text{sm}}(\cdot )$ and $f_{c}(\cdot |\Theta )$ stand for the soft-max activation function and the neural network classifier parameterized with Θ, respectively. With the one-hot encoded label $y_{i}$ in the source cohort, the cross-entropy loss is calculated as follows:


(2) $$l_{\text{CE}}=-\sum_{s\in S}y_{i}^{\left(s\right)}\;\text{log}\;(p_{i}^{\left(s\right)})$$

Meanwhile, the classifier adjusts its prediction with the guidance of prognostic information of each patient from the target cohort through an on-policy reinforcement learning algorithm, REINFORCE [24]. It samples a subtype prediction ${\hat{y}}_{i}$ of patient i from a multinomial distribution parameterized by the predicted subtype probability $p_{i}$ as follows:


(3) $${\hat{y}}_{i}∼P_{\text{multinomial}}(s|p_{i})$$

Such a random sampling strategy encourages the exploration of better classification boundaries and is only used for training. The subtype prediction for inference is deterministic with an argmax operation given predicted probabilities p. Then, for a specific subtype s, we calculate the restricted mean survival time (RMST) $\tau_{s}$. This calculation is based on two aspects related to each patient within the population of subtype s as shown Equation 4: one is the lifetime $t_{i}$ of the patient, and the other is the observation of the patient’s survival status, which we denote as $d_{i}$.


(4) $$\tau_{s}=f_{\text{RMST}}(\left\{(t_{i},d_{i}\right)|{ŷ}_{i}^{\left(s\right)}=1\})$$

The detailed calculation of $f_{\text{RMST}}$ is provided in latter sections. All RMST values are used to calculate a reward r to assess the prognostic discrimination of the predicted subtypes as follows:


(5) $$r=f_{\text{min}}(\{\tau_{a}-\tau_{b}|a,b\in \{1,\;2,\;\ldots ,\;k\},\;a<b\})$$

where $f_{\text{min}}$ outputs the minimal value from a set. Without the loss of generality, we use a smaller number to annotate the subtype with a better prognosis which is known a priori from the source cohort. However, since such behavior evaluation with rewards is usually noisy, a reward baseline is required for stabilization [25], which includes an input-dependent component $b_{i}$ and an input-independent component $b_{c}$, defined as follows:


(6) $$q=r-b_{i}-b_{c}$$

(7) $$b_{i}=f_{b}(x_{i}|\phi )$$

where $f_{b}$ represents a neural network predictor with ϕ as parameters. Both types of baseline components are adjusted by minimizing the baseline loss as follows:


(8) $$l_{\text{BL}}=\left.\|r-b_{i}-b_{c}\right.\|$$

where ‖·‖ is the L2 norm. Afterward, the predicted subtype probabilities, the sampled subtypes, and the baseline-stabilized rewards produce an approximation of the policy gradient [24,25] as follows:


(9) $$\nabla_{\theta }J\approx \nabla_{\theta }\frac{1}{N}\sum_{i=1}^{N}\;\text{log}\;(f_{c}(x_{i}|\theta ))q$$

where N is the number of patients. It regulates the classifier with the aim of improving its prognostic discrimination. The aforementioned objective can be implemented with a cross-entropy loss given the predicted probability p and the sampled prediction $\hat{y}$ as follows:


(10) $$l_{\text{RE}}=-\frac{1}{N}\sum_{i=1}^{N}\sum_{s\in S}{\hat{y}}_{i}^{\left(s\right)}\;\text{log}\;(p_{i}^{\left(s\right)})q$$

The final loss function for training the classifier is defined as:


(11) $$l=\omega_{1}l_{\text{CE}}+\omega_{2}l_{\text{RE}}$$

where $\omega_{1}$ and $\omega_{2}$ are coefficients between 0 and 1. Note that the aforementioned procedure describes the training phase of the classifier and there is no need for survival information during the inference phase.

The calculation of RMST is simple, but it requires the predicted subtyping results for all the patients in the mini batch, which are discrete and undifferentiable. Hence, we exploit the on-policy reinforcement learning algorithm REINFORCE by following the approach in [26] to approximate the true gradients and keep an end-to-end training procedure. Furthermore, the reason for using an on-policy algorithm is that it is simpler to implement compared to an off-policy algorithm. The latter needs more elaborate tuning of the algorithm such as replay buffer and the exploration strategy.

### Simulated dataset generation

**Figure 2:**
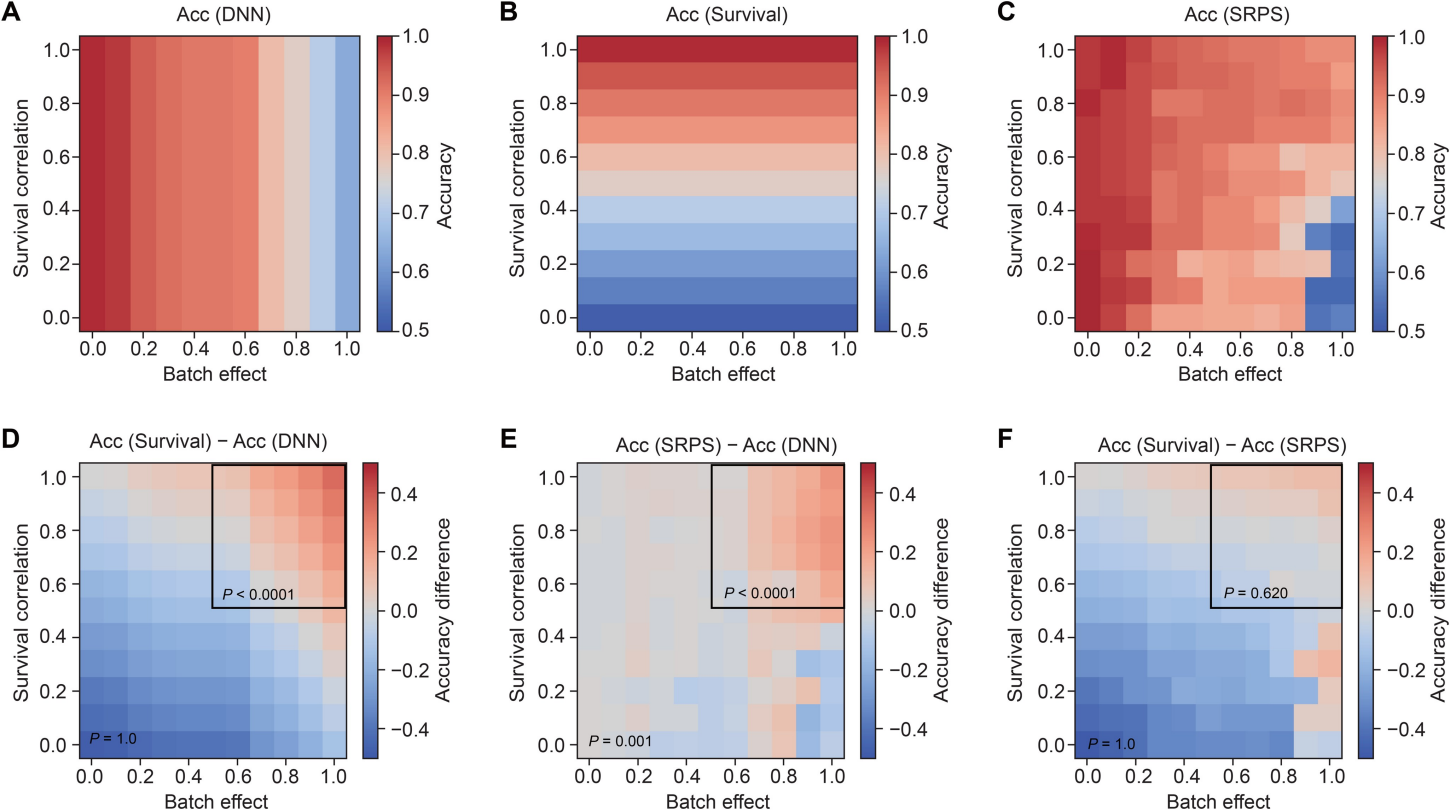
SRPS effectively improves classification accuracy by using prognostic information Each panel displays an accuracy map derived from 121 simulated toy datasets with varying levels of batch effect (between source and target cohorts) and survival correlation (between survival time and subtype) (see Method and Supplementary material for details). In each experiment, 20% of the samples from the target cohort are used for testing. The upper row exhibits the accuracy maps for three different subtyping strategies on the target cohort. **A**. Feature-based strategy. A DNN trained solely on features from the source cohort predicts two subtypes. **B**. Survival-based strategy. Patients in the target cohort are directly stratified by applying a 30-month threshold to their survival times. While this strategy is impractical in real life since survival time is unavailable for a certain patient who is still at risk, it is only used as a theoretical reference when such prognostic information could be fully exploited (or accurately predicted) for subtyping. **C**. SRPS. Our proposed algorithm uses features for prediction and is adjusted by prognostic information in the target cohort during training. **D**.–**F**. Residual accuracy maps obtained by element-wise subtraction between two strategies: Survival – DNN (D), SRPS – DNN (E), and Survival – SRPS (F). The one-tailed Student’s *t*-test was utilized to examine whether the values in the whole area or the upper-right area are significantly above zero. Acc, accuracy; DNN, deep neural network.

A total of 121 pairs of toy datasets were created to examine two basic assumptions about SRPS. The original dataset pair (Figure S1, top) consists of a source and a target cohort with identical features $x_{\text{origin}}$ whose values range between 0 and 1 and the feature dimension is 20. Normally distributed random noise $\sigma_{\text{noise}}$ was added to the features as batch effects between the source and target cohorts. The mean of the noise vector was uniformly sampled from [0,1] independently along each dimension, and the standard deviation (SD) was set to 0.2. The resulting feature vector is $x_{\text{origin}}+\alpha \sigma_{\text{noise}}$, where the noise weight α was gradually increased from 0 to 4 with a step of 0.4, corresponding to the lowest batch effect scale 0 to the strongest scale 1, respectively, as shown in **Figure 2**. The survival time of all patients was strictly divided according to the subtype labels, where S-I patients all survived longer than 30 months while S-II patients died before 30 months without any censoring for simplicity. To decrease the relative survival correlation between subtype and survival time, which leads to a weaker prognostic discrimination of subtypes, we randomly swapped the survival times of patients between two subtypes. The swapping ratio ranged from 0 to 0.2 with a step of 0.02, corresponding to the strongest relative correlation 1 and weakest relative correlation 0, respectively, as shown in Figure 2.

The simulated datasets for benchmarking (Figure S2) were generated with the R package Splatter [27], using the splatSimulate function. We generated two sets of datasets: one with batch effects and the other without. In each dataset, we simulated 1000 samples with three different subtypes, which were divided into two batches. The batch effect between batches was controlled by two parameters, batch.facLoc and batch.facScale, both of which were set to 0.01 for no batch effects and 2 for large batch effects. The feature size of all samples was set to 1000. The survival data were generated with the R package Simsurv [28], following the Weibull distribution with the coefficients (λ=0.005, γ=0.5). We carried out two experiments to validate the reliability of the simulated data. Figure S3 demonstrates that the distributional divergence between the simulated data and a real-world HCC cohort is comparable to the divergence between two real-world HCC cohorts. Furthermore, Figure S4 indicates that the difficulty of predicting patient prognosis, measured by the Concordance index (C-index) using a random survival forest model, between the simulated data and a real-world cohort is also comparable.

### Accuracy maps on toy datasets

We employed the aforementioned 121 pairs of toy datasets to examine the influence of batch effects between the source and target cohorts, as well as the correlation between subtype and survival time in the target cohort (termed survival correlation). The 121 pairs of datasets form a Cartesian product of 11 batch-effect levels and 11 survival-correlation levels. As shown in Figure 2, each cube in the accuracy maps represents the experiment result for a specific pair of source and target datasets, with axes indicating the batch-effect and survival-correlation levels (x-axis for increasing batch-effect levels, and y-axis for increasing survival-correlation levels).

For each experiment, the evaluation metric is prediction accuracy, defined as:


(12) $$\text{Prediction}\;a\text{ccuracy}=\frac{\text{Correct}\;p\text{redictions}}{\text{Sample}\;\text{size}}$$

where accuracy is calculated on 20% of the samples in the target cohort that were not used for model training. This definition is consistently applied to all experiments involving synthesized datasets.

Three accuracy maps (Figure 2A–C) were constructed for different subtyping strategies. (1) Feature-based strategy (Figure 2A): a deep neural network (DNN) trained on features from the source cohort predicts two subtypes. (2) Survival-based strategy (Figure 2B): patients are stratified by survival time using a threshold of 30 months. This strategy, while theoretical due to its reliance on unavailable survival data for at-risk patients, serves as a reference for fully exploiting prognostic information. (3) SRPS (Figure 2C): our proposed algorithm.

To further compare these strategies, three residual accuracy maps (Figure 2D–F) were generated by performing element-wise subtraction between the accuracy maps. For example, Figure 2D shows the result of subtracting the accuracy of the DNN-based strategy from that of the survival-based strategy across all 121 dataset pairs.

### Real-world proteomic data preprocessing

Prior to analysis, the raw proteomic expression matrix of each cohort was preprocessed. In these matrices, rows and columns represent samples and proteins, respectively. The preprocessing procedure contains the following steps: (1) each row of the profile matrix was normalized using quantile normalization; (2) proteins detected in less than 30% of samples were filtered out; (3) all missing values were imputed as zero; (4) each column was normalized with Z-score normalization; (5) only proteins commonly identified in both the source and target cohorts were retained; and (6) the resulting protein sets in two scenarios were then intersected with the signature protein set suggested by the source cohort’s literature, respectively.

Consequently, when transferring the subtypes from Jiang et al.’s HCC cohort [1] to other two HCC cohorts (*i.e.*, Gao et al.’s cohort [2] and Xing et al.’s cohort [29]) or to Xu et al.’s lung adenocarcinoma (LUAD) cohort [5], we used the signature proteins suggested by Jiang et al. [1], yielding feature dimension of 1097 for proteomic profile matrices (Table S1).

The raw proteomic profiles and associated clinical information can also be downloaded from the following publications: Jiang et al.’s HCC cohort (https://www.nature.com/articles/s41586-019-0987-8), Gao et al.’s HCC cohort (https://www.cell.com/cell/fulltext/S0092-8674(19)31003-7), Xing et al.’s HCC cohort (https://www.cell.com/cell-reports-medicine/fulltext/S2666-3791(23)00509-8), and Xu et al.’s LUAD cohort (https://www.cell.com/cell/fulltext/S0092-8674(20)30676-0).

### RMST

Given a series of paired survival time and status (dead or not) $\left\{(o_{1},u_{2}),\;(o_{1},u_{2}),\;...,\;(o_{n},u_{n})\right\}$ of n patients, we first estimate a Kaplan-Meier (KM) curve which consists of successive survival probabilities $s_{t}$ at each time within the time interval $t\in \{t_{0},t_{1},t_{2},\;\ldots ,\;t_{\text{max}}\}$. At $t=t_{0}$, we have $s_{0}=1$, and the consecutive probabilities can be extrapolated as follows:


(13) $$s_{t+1}=s_{t}\times (1-\frac{d_{t}}{n_{t}})$$

where $d_{t}$ denotes the number of subjects who died during the interval $\left[t_{k},t_{k}+1\right]$, and $n_{t}$ denotes the number of subjects living at the start. It should be noted that subjects who are lost to follow-up are regarded as censored and are not involved in $n_{t}$. RMST is calculated as the area under the KM curve within a restricted time interval, *i.e.*, from 0 to 60 months in our tests.

RMST is used because of its simplicity and directionality. When compared with other divergence metrics such as Kullback-Leibler, Maximum-Mean Discrepancy (MMD), and the Kuiper test that only measure the distance between the survival distributions of two subtypes as mentioned in [30], the difference in RMST between two subtypes is simple to calculate and clearly indicates which subtype holds a better prognosis. Therefore, it can largely boost the learning efficiency from the prognostic information.

### Training settings

The classifier of SRPS consists of a single linear layer with L1 regularization (coefficient = ϕ) and dropout (dropout rate is set to $\rho_{\text{su}}$ for supervised learning and $\rho_{\text{rl}}$ for reinforcement learning). The output dimension equals the number of subtypes. The baseline estimator consists of three layers with $n_{h}$ neurons in each hidden layer using the sigmoid activation function except the output layer. The output dimension is 1.

Both models are optimized with Adam optimizers given learning rates $\alpha_{\text{rl}}$ and $\alpha_{\text{bl}}$, respectively. The models are trained with the full batch for 10,000 episodes with or without using early stop (ε=true/false) which saves the model with the highest ΔRMST on validation set.

Hyper-parameters are manually selected according to the performance on the validation set within the possible combinations of following settings: $n_{h}=\{10,100\},\;\omega_{1}=\{0.1,0.01,0.001\},\omega_{2}=1,\;\phi =\{10^{-5},10^{-4}\},\;\rho_{\text{su}}=\{0,0.3,0.8\},\;\rho_{\text{rl}}=\{0,0.2\},\text{ε}=\{\text{true},\text{false}\}$. Given all possible combinations of hyper-parameters, we select the best model with different criteria on simulated and real-world datasets, respectively. For testing the model with simulated data, we apply the validation accuracy as the selection criterion. For testing the model on real-world datasets, we pick the model with the highest log-rank score for OS and an ssGSEA similarity score no less than 0.5 on the validation set, since there is no subtype label for the target dataset.

The model and training process was implemented in Python using the Tensorflow library and was deployed on a server equipped with an InteI XII Gold 6242R CPU and a Quadro GV100 GPU. Each run of training lasted about 2 min.

### ssGSEA similarity

For each patient in the real-world proteomic datasets, functional enrichment analysis [31] was performed using the ssGSEA algorithm implemented in the R package GSVA [32]. The quantile-normalized protein expression matrix was input to GSVA to calculate the ssGSEA scores for gene sets with at least ten overlapping genes. The gene set of interest was provided in [1,5]. After obtaining the enrichment scores for the gene set of interest of each patient, we calculated the average enrichment score for each subtype and estimated the similarity between cohorts with the cosine similarity. The final similarity score between the two cohorts is the average similarity of all subtypes.

### Log-rank score and C-index

The log-rank score was defined as $-\text{lo}g_{10}\;P$ based on the *P* value of a log-rank test [33]. This score indicates the likelihood that all groups share the same lifetime distribution. A higher score means better prognostic discrimination. C-index [34] calculates the fraction of pairs of subjects for which the model correctly predicts the order of survival times while also considering censoring. A C-index of 0.5 indicates random prediction, while 1.0 means perfect prediction. It was calculated by checking the concordance between the survival time and probability difference between subtypes with the best and worst prognoses, *i.e.*, $p_{i}^{\left(1\right)}-p_{i}^{\left(3\right)}$. Here, $p_{i}$ denotes the predicted probability vector of patient i, with $p_{i}^{\left(1\right)}$ and $p_{i}^{\left(3\right)}$ corresponding to the probabilities of being subtype S-I (best prognosis) and subtype S-III (worst prognosis), respectively.

### Five-fold cross-validation

All benchmarking results (except for testing on toy datasets) are reported based on a 5-fold cross-validation repeated 5 times with different random seeds. More concretely, for each repeat, we set the random seed and evenly split the data into 5 folds. Then a loop starts by selecting 3 folds for training, one fold for validation, and the remaining fold for testing. The loop continues until all 5 folds have been used as the test fold, and the testing results of all 5 folds are regarded as the final testing result of the current repeat.

### Implementation of competitive algorithms

The random forest (RF) algorithm is an ensemble of tree predictors where all trees vote for the most likely class prediction. We used the implementation from the Python package sklearn [35] with the function RandomForestClassifier, setting the maximum depth to 10 and training it as a classification model under the supervision of subtyping labels provided in the source cohort.

DNN is a neural network consisting of a hidden layer, a dropout layer, and a prediction layer, and was trained purely in a supervised manner. It was implemented with the TensorFlow library in Python. The hidden dimension and dropout rate were set to 20 and 0.8, respectively, to avoid overfitting. The cross-entropy loss and Adam optimizer were utilized. The learning rate was set to 0.01, and the model was trained for 2000 epochs.

Harmony is an algorithm designed to remove batch effects in single-cell data by projecting data from various batches into a batch-invariant representation space through soft clustering [13]. Then, samples can be classified with RF within this space without being affected by batch effects. We first performed embeddings of the raw profile matrix with principal component analysis (PCA) to obtain the low-dimensional representations (100 or 10 dimensions) of the data, and then applied the Python implementation of the Harmony algorithm from the package harmony-py with its default parameter settings.

Deep Adversarial Neural Network (DANN) is a counterpart solution in machine learning and computer vision that addresses the domain adaptation task. Although there are several more state-of-the-art (SOTA) algorithms for image domain adaptation [17,36,37], we found they could not lead to any improvement potentially due to the lack of effective neural network structure for encoding omics data (Figure S5). DANN was implemented with the Python TensorFlow library. It includes an encoder $z=f_{\text{encode}}(x)$, a task classifier $\hat{y}=f_{\text{task}}(z)$, and a domain classifier $\hat{d}=f_{\text{domain}}(z)$. The task classifier was trained to minimize the cross-entropy loss between subtype predictions and labels, while the domain classifier was optimized to correctly predict the domain of the sample (*i.e.*, whether the sample was drawn from the source or target cohort). The critical trick was to insert a layer that can reverse the gradients between the domain classifier and the encoder, which encourages the encoder to learn domain-invariant features to confuse the domain classifier. The number of layers in the encoder and classifiers were tuned with two options (1 and 2). The hidden dimensions of the encoder and classifiers were set to 20 and 10, respectively. The learning rate was set to 0.01, and the dropout rate was tuned with two options (0 and 0.3).

DeepCLife [30] is a deep learning-based approach that solves an unsupervised clustering problem with survival information. For fairness, its classifier was optimized by minimizing the original survival loss on the target cohort together with the supervised loss on the source cohort. We referred to this adapted version as semi-deepCLife, considering it to be the most relevant baseline method for SRPS. We reimplemented the TensorFlow version according to the original open-source code available at https://github.com/PurdueMINDS/DeepLifetimeClustering, which is based on the PyTorch library. The parameters tuned for semi-deepCLife are learning rate = {0.01, 0.001}, L1 regularization = $\left\{10^{-5},10^{-4}\right\}$, dropout rate = {0.3, 0.8}, and weight of survival_loss = {0.01, 0.05}.

### Ablated models of SRPS

The first ablated model removes the baseline predictions of rewards in SRPS. It modifies Equation 6 to q=r and stops optimizing the baseline predictor. The second ablation replaces the reinforcement learning approach with a soft relaxation strategy, as used in deepCLife. The main difference is that instead of applying the Kuiper test *P* value-based loss function, we calculate and maximize a soft version of ΔRMST for optimization. More concretely, when calculating $d_{t}$ and $n_{t}$ in Equation 13 for a specific subtype group, the contribution of each subject is weighted by the probability that the subject belongs to that subtype. Such an operation keeps the entire tensor graph differentiable and allows direct optimization through the back-propagation algorithm.

### Definition of subtype significance score

Given the parameter matrix of the classifier in SRPS as $\Theta \in R^{k\times d}$, the logits vector $l\in R^{k}$ based on an input profile vector $x\in R^{d}$ is formulated as l=Θ**x**. The predicted probability vector is:


(14) $$p=\frac{\;\text{exp}\;\left(l\right)}{\sum_{s}\;\text{exp}\;\left(l^{\left(s\right)}\right)}$$

where d and k denote the profile dimension and the number of subtypes, respectively, and $l^{\left(s\right)}$ is the *s*-th element in the vector l. Unlike the random sampling based on predicted probabilities during training, the subtype prediction in the inference stage is deterministic using the argmax operation, as illustrated in Figure 1. In other words, for a,b ∈{1, 2, …, k} where b≠a, the predicted subtype s will be a if and only if $p^{\left(a\right)}>p^{\left(b\right)}\;\Rightarrow \;l^{\left(a\right)}>l^{\left(b\right)}\;\Rightarrow \Theta^{\left(a\right)}x>\Theta^{\left(b\right)}x$ holds true for any possible b, where $\Theta^{\left(a\right)}$ represents the *a*-th row vector of Θ. Therefore, when we only focus on a specific feature $x^{\left(i\right)},\;i\in \{1,\;2,\;\ldots ,\;d\}$, we can approximately have:


(15) $$P(s=a|x^{\left(i\right)})\propto P(\Theta^{\left(a,i\right)}-\Theta^{\left(b,i\right)}>0|x^{\left(i\right)})$$

where a,b∈{1, 2, ..., k} and b≠a. $\Theta^{\left(a,i\right)}$ represents the element at the *a*-th row and *i*-th column of Θ. Equation 15 indicates that the probability of predicting the given profile as subtype a when only considering a single protein expression $x^{\left(i\right)}$ is proportional to its corresponding weight $\Theta^{\left(a,i\right)}$ and inversely proportional to the weights of other subtypes. Accordingly, we give the following definition about Δweight:


(16) $$\Delta \text{weigh}t^{\left(a,i\right)}=\Theta^{\left(a,i\right)}-\frac{\sum_{b\neq a}\Theta^{\left(b,i\right)}}{k-1}$$

Since this metric is defined based on model weights and may be affected by random initialization, we utilized the average value of multiple models to identify significant proteins. The convergence of this approach during training and its stability with varying numbers of models are shown in Figures S6 and S7.

### Cell culture and small interfering RNA transfection

HCC cell lines Huh7, Hep3B, MHCC-LM6, and MHCC-97H were cultured in Dulbecco’s Modified Eagle Medium (DMEM; Catalog No. C11995500BT, Gibco, Grand Island, NY) supplemented with 10% fetal bovine serum (FBS; Catalog No. FBS-CS500, Newzerum, Christchurch, New Zealand), 100 U/ml penicillin (Catalog No. 15140-122, Gibco), and 100 μg/ml streptomycin (Catalog No. 15140-122, Gibco). All cells were incubated at 37°C with 5% CO_2_. Cells were seeded in 6-well plates before transfection. When confluence approached 50%, cells were transfected twice with the indicated small interfering RNAs (siRNAs) at 24-h intervals using TurboFect Transfection Reagent (Catalog No. R0531, Thermo Fisher Scientific, Waltham, MA). Cells were collected 48 h after transfection for mRNA expression assay. The siRNA sequences targeting *PPIC* were as follows: si-*PPIC*-1 (5′-GCUCUAGCAACAGGAGAGA-3′) and si-*PPIC*-2 (5′-CUCGAUCAUCAACAGUGGC-3′).

### mRNA expression assay

Total RNA was extracted using TRIzol Reagent (Catalog No. 15596018CN, Invitrogen, Carlsbad, CA) according to the manufacturer’s instructions. cDNA was synthesized using HiScript III All-in-one RT SuperMix Perfect (Catalog No. R333-01, Vazyme, Nanjing, China). mRNA expression was measured by quantitative real-time PCR (qPCR) using Taq Pro Universal SYBR qPCR Master Mix (Catalog No. Q712-02, Vazyme) on a Bio-Rad CFX96 instrument. Statistical graphs were generated using GraphPad Prism (v8.3.0). The qPCR primers used in this study were as follows: *ACTIN*-Forward (5′-CACCATTGGCAATGAGCGGTTC-3′), *ACTIN*-Reverse (5′-AGGTCTTTGCGGATGTCCACGT-3′), *PPIC*-Forward (5′-CTGCTGCTACCTCTCGTGC-3′), and *PPIC*-Reverse (5′-GCCAATCACAATTCTGCCAACA-3′).

### Cell viability and colony formation assays

For cell viability assay, after the indicated treatments, approximately 5 × 10^3^ cells per well were seeded into 96-well plates. Cell viability was monitored daily for five days using the CCK8 kit (Catalog No. AQ308, Aqlabtech, Beijing, China), with the optical density (OD) at 450 nm measured each day on a Spark Microplate Reader (TECAN). For cell colony formation assay, after the indicated treatments, approximately 5 × 10^3^ cells per well were seeded into 6-well plates. After 12 days of culture, cells were fixed with methanol and stained with Crystal Violet Staining Solution (Catalog No. C0121, Beyotime, Shanghai, China) for 1 h. After washing three times with phosphate-buffered saline (PBS), colonies were photographed. Statistical graphs were generated using GraphPad Prism (v8.3.0).

### Cell migration assay

After the indicated treatment, 1 × 10^5^ cells were resuspended in 200 μl of FBS-free DMEM (Catalog No. C11995500BT, Gibco) and seeded onto the upper chamber of a Transwell filter with 8-µm pores. The lower chamber contained 500 μl of DMEM supplemented with 20% FBS (Catalog No. FBS-CS500, Newzerum). After 48-h incubation, cells on the underside of the filter were fixed with methanol and stained with Crystal Violet Staining Solution (Catalog No. C0121, Beyotime) for 1 h. After washing, photos were taken by a microscope equipped with a digital camera. Statistical graphs were generated using GraphPad Prism (v8.3.0).

## Results

### SRPS improves the classification accuracy for prognosis-discriminative subtypes in the presence of inter-cohort batch effects

SRPS aims at improving subtyping accuracy by using the correlation between subtype and survival time through deep reinforcement learning. It is based on two assumptions: (1) feature-based subtyping can be improved by exploiting prognostic information; and (2) prognostic information can be effectively exploited through deep reinforcement learning.

To validate these assumptions, 121 pairs of toy datasets (Figure S1) were simulated, and three different patient stratification strategies were compared in varying levels of batch effect between source and target cohorts and survival correlation (the correlation between subtype and survival time). The performance of the feature-based stratification strategy based on supervised learning (DNN) was only affected by the batch effect (Figure 2A). On the contrary, the accuracy of the survival-based stratification method (Survival), which divided patients at a lifetime threshold of 30 in the target cohort, was only related to the survival correlation (Figure 2B). SRPS (Figure 2C) achieved more robust performance even with strong batch effects or low survival correlation due to the integration of both feature and prognostic information.

Residual accuracy maps between two strategies showed that the survival-based method significantly outperformed DNN within the upper-right region (large batch effects and high survival correlation; *P* < 0.0001) (Figure 2D), proving that prognostic information alone can improve classification accuracy in such conditions. By contrast, SRPS significantly surpassed DNN not only within the upper-right region (*P* < 0.0001) but across the entire space (*P* = 0.001) (Figure 2E). Furthermore, SRPS performed comparably to the survival-based method within the upper-right region (*P* = 0.620) (Figure 2F), suggesting that SRPS can highly exploit the prognostic information through deep reinforcement learning.

### SRPS outperforms competitive methods and ablated models on simulated datasets

We evaluated the SOTA performance of SRPS on two pairs of simulated datasets (Figure S2) by comparing it with five competitive methods: RF [11] and DNN [12] for supervised learning, RF combined with Harmony (RFH) [13] for batch effect removal, DANN [16] for domain adaptation, and semi-deepCLife [30] for a prognosis-guided approach. A model ablation study was also carried out by either removing a certain component of reinforcement learning [*i.e.*, SRPS (no baseline)] or replacing reinforcement learning with an alternative approach [*i.e.*, SRPS (soft)]. The evaluation metrics include classification accuracy, ssGSEA similarity, log-rank score, and C-index. All metrics are presented as mean ± SD based on a 5-fold cross-validation repeated 5 times.

#### Comparison with baseline methods

**Figure 3:**
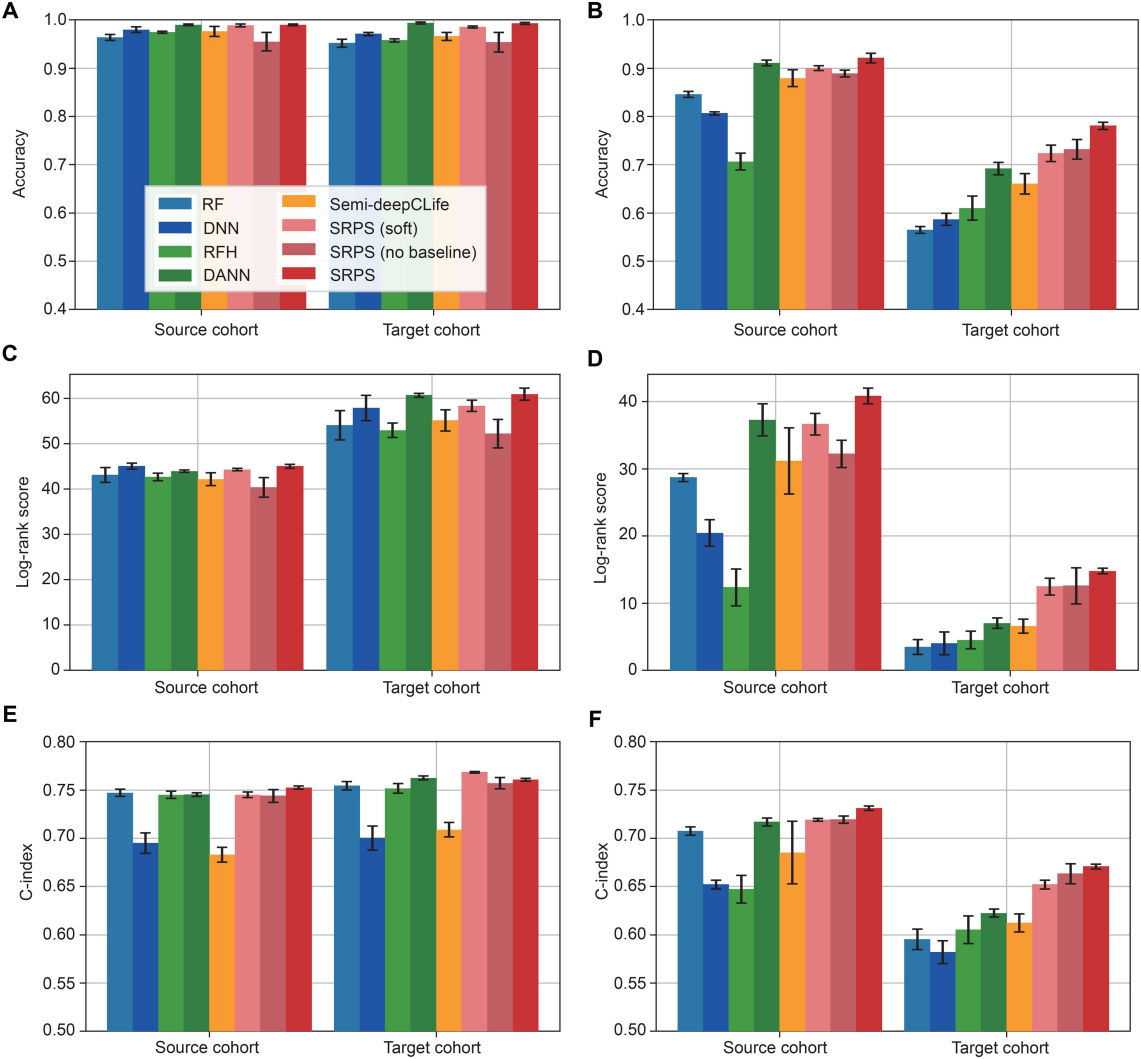
SRPS outperforms competitive methods and ablated models on simulated datasets Bar plots show the benchmarking results on two simulated datasets without batch effects (**A**, **C**, and **E**) and with batch effects (**B**, **D**, and **F**). Performance metrics (mean ± SD) are reported based on a 5-fold cross-validation test repeated five times. The competitive methods are: (1) RF, (2) DNN for supervised learning, (3) RFH for batch effect removal, (4) DANN for domain adaptation, and (5) semi-deepClife for a prognosis-guided approach. SRPS (soft) and SRPS (no baseline) are ablated models of SRPS. SD, standard deviation; C-index, Concordance index; RF, random forest; RFH, RF combined with Harmony; DANN, deep adversarial neural network.

When tested on datasets without batch effects, all methods achieved high classification accuracy (above 0.9) on both the source and target cohorts, showing their generalization ability on samples drawn from a distribution similar to the training data (**Figure 3**A). However, their performance degenerated to varying degrees when batch effects existed (Figure 3B). As pure supervised learning methods, RF and DNN showed an expected accuracy drop on the target cohort with a biased distribution drift. RFH outperformed RF and DNN on the target cohort but displayed a notable accuracy drop on the source cohort. This is likely because its attempt to remove batch effects diminishes the biological features of each subtype, which may partially stem from the fact that it does not employ the labels in the source cohort. DANN was the most powerful baseline approach, but it still fell behind SRPS, especially on the target cohort. Although there are other domain adaptation approaches implementing more sophisticated learning mechanisms such as conditional domain adversarial network (CDAN) [36] and maximum classifier discrepancy (MCD) [17], they did not achieve higher subtyping accuracy in our tests (Figure S5). Semi-deepCLife, the most similar competitive algorithm to SRPS, performed only comparably to DANN. This might be attributable to two reasons: (1) the survival loss of deepCLife does not specify which subtype should have a superior prognosis, which may incur conflicts with the label-based supervision loss during training; and (2) the lack of random exploration in stochastic gradient descents with a large batch, which is required for accurate estimation of lifetime distributions. Therefore, the straightforward combination of deepCLife and supervised learning is not an effective solution. In contrast, SRPS substantially outperformed all competitive methods when batch effects existed between cohorts. More specifically, it improved the target domain accuracy by 12% compared to the second-ranked model, DANN.

As for the log-rank score, all methods achieved high performance on datasets without batch effects (Figure 3C), accurately predicting subtypes which were strongly correlated to prognosis in both the source and target cohorts. However, their performance decreased remarkably on the target cohort with large batch effects (Figure 3D). As expected, SRPS enhanced the log-rank score by more than 100% compared to the best baseline, DANN. Furthermore, SRPS exhibited similar superior trends in terms of C-index (Figure 3E and F).

#### Model ablation study

We conducted an ablation study to evaluate the contribution of two critical reinforcement learning components. First, we removed the reward baseline estimator, which is important for gradient stabilization in many policy gradient-based reinforcement learning algorithms [25]. The gradients estimated by REINFORCE [24] were calculated from random samples and caused noisy rewards. As shown in Figure 3, SRPS (no baseline) displayed higher SD in accuracy, log-rank score, and C-index on the target cohort compared to the full SRPS method, indicating the high variance during the learning procedure of this ablated model. Second, we replaced the entire reinforcement learning component with soft relaxation used in deepCLife, which is an alternative approach for dealing with non-differentiable components in a neural network. However, this substitution introduced the dilemma of batch size selection during training as mentioned above. As a result, this design severely compromised the performance of SRPS (Figure 3).

### SRPS outperforms competitive methods in real-world cohort adaptations of proteomic subtypes

To further demonstrate the SOTA performance of SRPS, we evaluated it against competitive methods in two different cohort adaptation scenarios with three HCC cohorts and an LUAD cohort. Each cohort provided proteomic profiles of all samples alongside clinical outcomes including OS and RFS.

**Figure 4:**
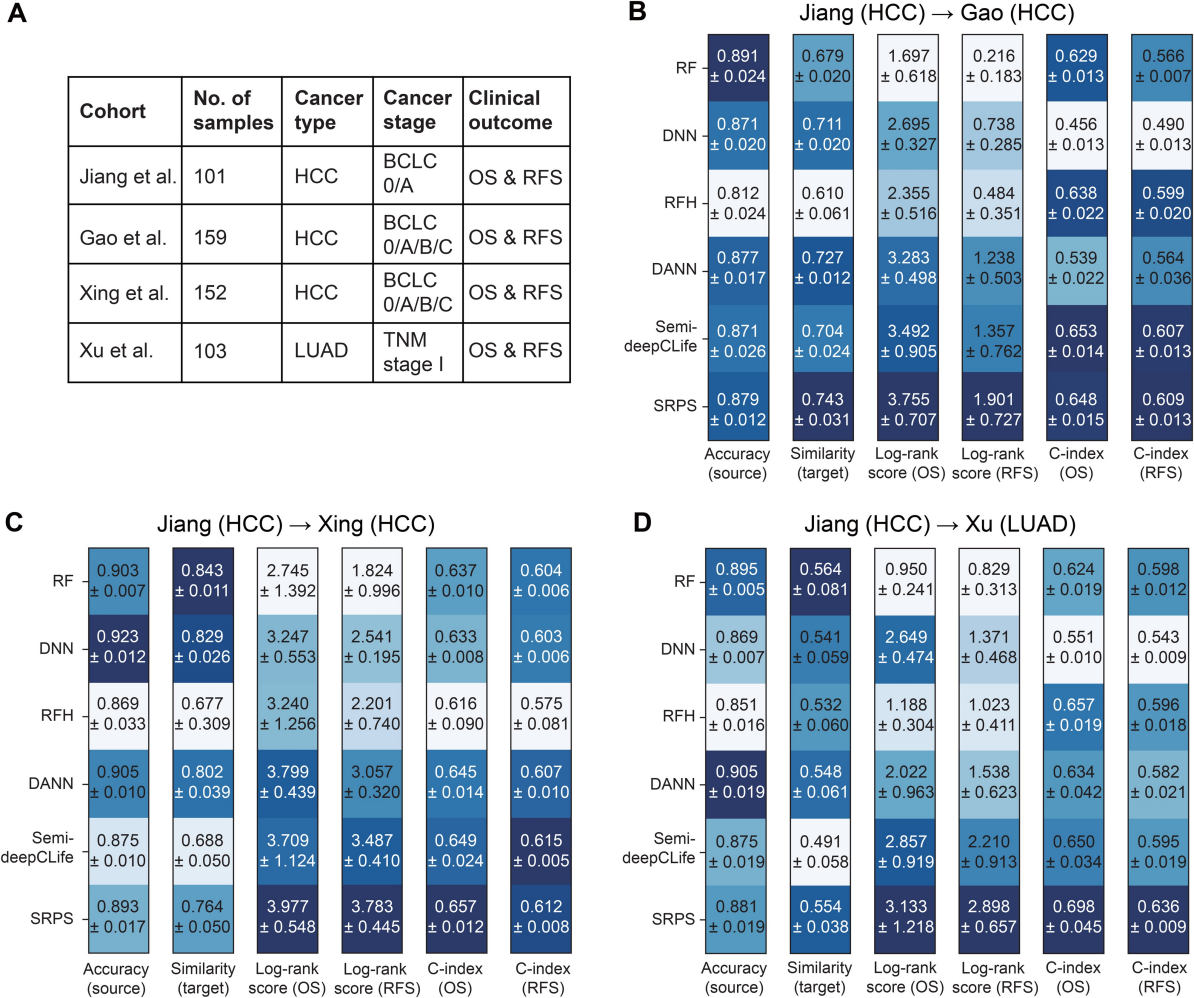
SRPS outperforms competitive methods on real-world cohort datasets **A**. Summary of the four real-world cohorts, including cancer type, number of samples, cancer stage, and clinical outcomes evaluated. **B**.–**D**. Performance of SRPS and competitive methods for transferring the subtypes discovered in the Jiang et al.’s cohort to the Gao et al.’s cohort (B), Xing et al.’s cohort (C), and Xu et al.’s cohort (D), respectively. Metrics include the accuracy in the source cohort, ssGSEA similarity of subtypes between the source and target cohorts, log-rank score for OS, log-rank score for RFS, C-index for OS, and C-index for RFS. Larger values represent better performance, as indicated by deeper colors in each column. HCC, hepatocellular carcinoma; LUAD, lung adenocarcinoma; OS, overall survival; RFS, recurrence-free survival; BCLC, Barcelona Clinic Liver Cancer; TNM, Tumor-Node-Metastasis; ssGSEA, single-sample gene set enrichment analysis.

The source cohort from Jiang et al. [1] comprised 101 Chinese HCC patients at early stages (0–A) based on the Barcelona Clinic Liver Cancer (BCLC) staging system [38]. According to the original study [1], all patients in this source cohort were classified into three subtypes with distinct proteomic features and prognostic outcomes, as shown in Figure S8. These subtype labels were then transferred to the target cohorts as prior knowledge. The target cohorts included: the Gao et al.’s cohort [2] (159 Chinese HCC patients, BCLC stages 0–C), the Xing et al.’s cohort [29] (152 Chinese HCC patients, BCLC stages 0–C), and the Xu et al.’s cohort [5] [103 Chinese LUAD patients, Tumor-Node-Metastasis (TNM) stage I] (**Figure 4**A). As shown in Figure S9, the proteomic profile matrices of these cohorts exhibited large inter-cohort heterogeneity.

#### Transferring HCC subtypes to other HCC cohorts with broader BCLC stages

We first transferred the proteomic subtypes of HCC patients from the Jiang et al.’s cohort to the Gao et al.’s and Xing et al.’s cohorts, respectively. The results are shown in Figure 4B and C. Except for RFH, all methods achieved similar prediction accuracy on the source cohort. Deep learning-based methods (DNN, DANN, semi-deepCLife, and SRPS) showed higher ssGSEA similarities of the classified subtypes between source and target cohorts, indicating that neural networks learn more generalizable features of each subtype. A similar trend was observed for the log-rank score on OS. Pure supervised learning or domain adaptation techniques (*e.g.*, DNN and DANN) performed poorly on the log-rank score for RFS, mainly due to the lack of significant differences in the RFS distributions of all subtypes in the source cohort. Consequently, this limitation was naturally inherited in their predictions on the target cohort. Semi-deepCLife was also designed to improve discrimination through exploiting two kinds of survival information together, and performed comparably to SRPS in C-index. Ultimately, SRPS exhibited powerful discriminative ability in terms of both OS and RFS with comparable classification accuracy on source cohort and ssGSEA similarity between two cohorts. Representative results of SRPS among five repeated tests on the Gao et al.’s cohort and Xing et al.’s cohort are shown in Figures S10 and S11. It is worth noting that clinically acceptable prognostic discrimination should reach a log-rank score above $-\log_{10}\;0.05\approx 1.3$.

#### Transferring HCC subtypes to a LUAD cohort

Next, we transferred subtypes from the Jiang et al.’s HCC cohort to the Xu et al.’s LUAD cohort (Figure 4D). SRPS again exhibited the highest prognostic discrimination for both OS and RFS among all methods, demonstrating its superiority in optimizing prognostic discrimination of subtypes. However, the ssGSEA similarities of all methods are lower than those observed in HCC-to-HCC transfers. It is reasonable, since the onset of cancer in different organs can have drastically heterogeneous biological processes. Thus, HCC and LUAD patients with consistent prognoses are unlikely to share similar proteomic patterns between their tumor samples. This observation suggests that although SRPS enhances the detection of prognostically consistent subtypes between two cohorts, it makes a compromise by reducing the biological consistency when the original subtypes are unlikely to exist naturally in the new cohort. Its discoveries adhere to the underlying biological facts. A representative result of SRPS among five repeated tests on the Xu et al.’s cohort is shown in Figure S12.

### PPIC is identified as a significant protein through model interpretation using subtype significance score

The interpretability of models is of importance in biomedical research, where a common solution is to keep the model as simple as possible. Therefore, the classifier utilized in SRPS for real-world proteomic data is a single-layer neural network with a parameter matrix shaped as k×d and is activated by a soft-max layer to generate probabilistic outputs. Based on this simple architecture, we proposed a subtype significance metric named Δweight to quantify the contribution of each protein to classifying patients into a specific subtype. Notably, the expression of each protein was processed with Z-score normalization, ensuring that each protein’s contribution to subtyping is independent of its original expression range. The total number of Δweight values equals to the number of values in the parameter matrix. A detailed definition of Δweight is provided in Method.

Based on the results of transferring HCC subtypes from the Jiang et al.’s cohort to the Gao et al.’s cohort, we investigated the weight matrices of all 25 classifier models of SRPS (5 random repeats × 5 folds), and focused on the subtype S-III of HCC patients that is associated with the worst prognosis after surgery. The average Δweight across all 25 models was used in the following analysis to alleviate the influence of random initialization. Additionally, since Δweight is defined based on model weights which vary during training, we carried out stability experiments. Figure S6 shows that the ranking of the top 10 proteins based on the average Δweight becomes stable quickly during training. Furthermore, the relationship between ranking stability and the number of models required is shown in Figure S7.

#### SRPS classifies patients with prognosis-discriminative features

**Figure 5:**
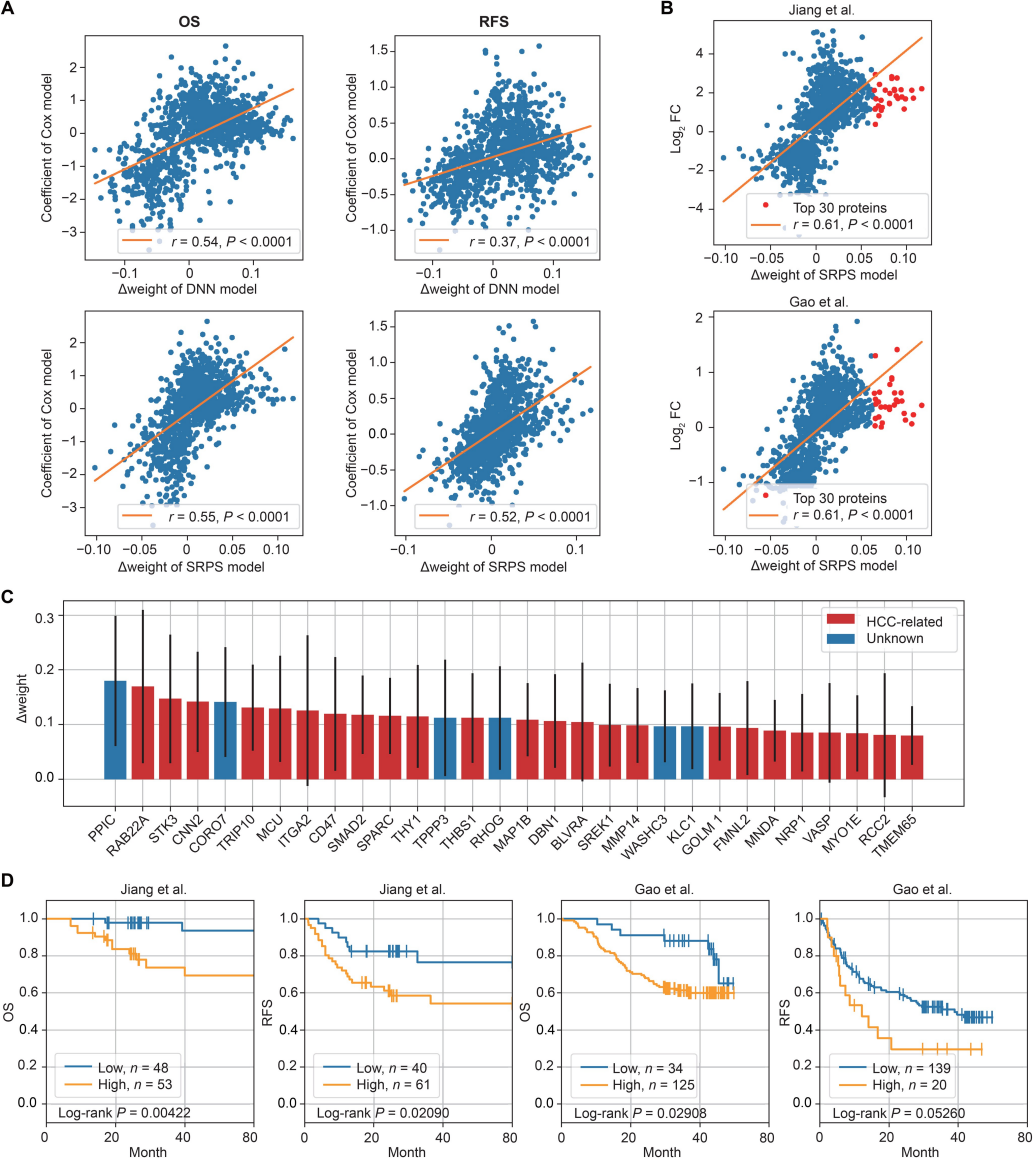
PPIC was identified as a significant protein through model interpretation using subtype significance score **A**. Correlation between the prognostic discriminative power (coefficient of a univariate Cox model) and the subtype significance score (Δweight) of each protein in the DNN and SRPS models in terms of OS and RFS. **B**. Correlation between the subtype significance score (∆weight) of each protein in the SRPS model and the corresponding log_2_ FC value in the Jiang et al.’s cohort (top) and the Gao et al.’s cohort (bottom). Red dots represent proteins with the top 30 subtype significance scores. **C**. Bar plot showing the subtype significance scores (mean ± SD) of the top 30 proteins. Color coding denotes whether the proteins have been formally reported as HCC-related (red) or not (blue) in the literature. **D**. Prognostic discriminative power of PPIC for OS and RFS on the Jiang et al.’s and Gao et al.’s cohorts. The threshold for dividing high- and low-expression groups in each plot is independently set to the value yielding the highest log-rank test significance. PPIC, peptidyl-prolyl *cis*-*trans* isomerase C; FC, fold change.

We first evaluated the correlation between the subtype significance score (Δweight) of the SRPS classifier and the prognostic discriminative power of each protein in the Gao et al.’s cohort. The prognostic discriminative power of each protein was measured by the coefficient of a univariate Cox regression model, and the correlation was estimated by the linear regression. For OS, the Δweight of SRPS showed a correlation (*r* = 0.55) similar to that of DNN (*r* = 0.54) (**Figure 5**A), mainly because DNN has already achieved good discrimination in OS (Figure 4B). In contrast, SRPS showed a much higher correlation (*r* = 0.52) than DNN (*r* = 0.37) for RFS (Figure 5A), as it enhanced the discrimination of RFS by a large margin (Figure 4B). This result suggests that SRPS improves the prognostic discrimination of subtypes by allocating higher weights to features that are more discriminative in prognosis during training, compared to the method without exploiting survival information.

#### SRPS discovers signature proteins beyond differential expression analysis

Differential expression analysis (DEA) is a common approach for identifying subtype-specific signature proteins and usually serves as a clue for biomarker discovery [1,2]. Hence, we calculated the correlation between Δweight and log_2_ fold change (an essential metric for DEA) in protein expression for subtype S-III in the Jiang et al.’s and Gao et al.’s cohorts (Figure 5B). While a positive correlation was observed in each cohort, obvious discrepancy was noted for the most significant proteins: the top 30 proteins ranked by Δweight did not correspond to those with the highest log_2_ fold change values. This observation suggests that the subtype significance score can uncover signature proteins potentially overlooked by classical DEA-based approaches.

#### PPIC identified by SRPS is an under-explored HCC-related protein and shows prognostic discriminative ability

We further investigated whether the 30 top-ranked proteins or their corresponding genes have been previously reported to be associated with HCC in the literature (Figure 5C). The results showed that 24 of these 30 proteins had literature evidence to support their relevance with HCC progression, indicating that SRPS classifies patients based on biologically meaningful features. Among the six under-explored HCC-related proteins, limited functional evidence has been reported for Coronin-7 (CORO7) and WASH complex subunit 3 (WASHC3) in cancer. However, PPIC, Tubulin polymerization-promoting protein family member 3 (TPPP3), Rho-related GTP-binding protein RhoG (RHOG), and Kinesin light chain 1 (KLC1) have been reported to play roles in cancer progression (*e.g.*, cell proliferation and metastasis) in other cancer types (Table S2), suggesting their potential involvement in HCC. More importantly, we found that PPIC, which had the highest subtype significance score, has received little attention in HCC-related research, thus triggering our deeper investigation into this protein.

Survival analyses in the Jiang et al.’s cohort and Gao et al.’s cohort for both OS and RFS were performed by stratifying patients based on PPIC expression. The results demonstrate the prognostic discriminative ability of PPIC for these two clinical outcomes (Figure 5D). For each analysis, the optimal threshold was independently determined by grid search, selecting the value that results in the highest significance of log-rank test. Therefore, PPIC effectively discriminates prognosis and shows potential as a biomarker for identifying HCC patients with poor survival.

### PPIC functions as a pro-cancer protein in HCC

**Figure 6:**
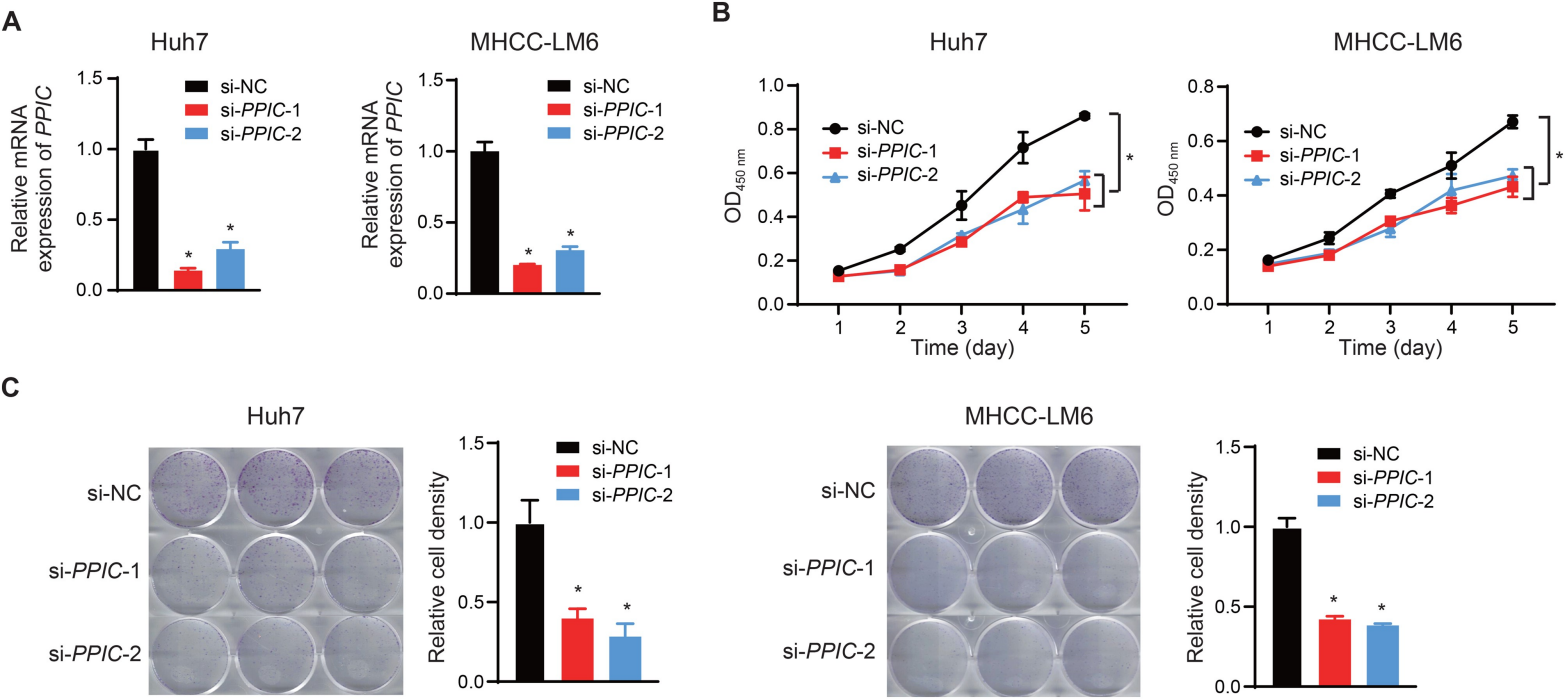
PPIC promotes cell proliferation and colony formation in HCC cell lines **A**. Relative mRNA expression of *PPIC* in Huh7 and MHCC-LM6 cells after transfection with indicated siRNAs. **B**. Cell proliferation curves of Huh7 and MHCC-LM6 cells after transfection with indicated siRNAs, measured by CCK-8 assay. **C**. Representative images (left) and quantification (right) of colony formation assays in Huh7 and MHCC-LM6 cells after transfection with indicated siRNAs. Data are presented as mean ± SEM. *, *P* < 0.05 (two-tailed Student’s *t*-test). siRNA, small interfering RNA; SEM, standard error of the mean.

Although PPIC has been reported to be highly expressed in several cancers, its role in HCC remains elusive. To determine the potential functions of PPIC in HCC, we performed siRNA-mediated knockdown of *PPIC* in multiple HCC cell lines (Huh7, MHCC-LM6, Hep3B, and MHCC-97H). As shown in **Figure 6**A and Figure S13A, the *PPIC* mRNA expression was significantly reduced in cells transfected with the siRNAs targeting *PPIC*. Notably, *PPIC* knockdown profoundly inhibited cell proliferation and colony formation in Huh7 and MHCC-LM6 cells (Figure 6B and C), which suggests that PPIC promotes HCC progression. Similarly, *PPIC* knockdown also significantly inhibited colony formation in Hep3B and MHCC-97H (Figure S13B). Additionally, *PPIC* knockdown strongly inhibited cell migration in Huh7 cells (Figure S13C). Collectively, these results indicate that PPIC functions as a pro-cancer protein in HCC, consistent with the results from SRPS. The underlying molecular mechanism needs further investigation.

## Discussion

Multicenter studies are essential in clinical oncology research to eliminate the biological and environmental biases inherent in sampled populations. Therefore, to transfer the practical value of PDPM to clinical applications, it is imperative to expand and validate discoveries from a single cohort across multiple independent cohorts. However, since proteins are the minimal functioning units of the human body system, the proteome provides a more real-time reflection of disease progression compared to the genome and transcriptome, therefore exhibiting substantial data heterogeneity across cohorts.

We propose the SRPS algorithm to adapt known proteomic subtypes from a labeled source cohort to an unlabeled target cohort. Unlike existing batch effect removal or domain adaptation techniques which seek indistinguishable representations between batches/domains with the risk of ignoring essential biological differences, SRPS unifies classification features between cohorts by preserving the prognostic discrimination of subtypes, a critical consideration for clinical applications. Through experiments on simulated and real-world data, we demonstrate the effectiveness of guiding the subtyping with survival information through reinforcement learning.

Deep reinforcement learning has achieved great success in optimizing neural networks through self-defined reward functions by encouraging desired behaviors and penalizing undesired ones without requiring explicit labels, as seen in gaming [39], robotics [40], and drug design [41]. It also provides a solution to estimate gradients of non-differentiable evaluations of the predictions [26], with soft approximation [30] or relaxation [42] serving as an alternative. By comparing SRPS with semi-deepCLife or SRPS (soft), we observed SRPS’s higher performance in prognostic discrimination. Moreover, this strategy can be applied to any objective due to the high degree of freedom in the reward function design. For instance, it is possible to reinforce the ssGSEA similarity of the subtypes between two cohorts by incorporating it into the reward function.

Since survival information has proven effective in guiding the learning process of subtyping, sophisticated neural network architectures become unnecessary to achieve robust performance. This allows for the use of a single dense layer in the classifier, which brings simplicity in model interpretation and alleviates overfitting. Consequently, PPIC was discovered as a critical protein for identifying HCC patients with poor prognosis.

Here, we demonstrate that PPIC is required for HCC cell proliferation and colony formation, indicating its pro-cancer functions. Although PPIC has been reported to be involved in endoplasmic reticulum redox homeostasis and hepatic stellate cell activation [43,44], its key molecular functions and direct substrates in HCC remain to be further elucidated. Thus, our results demonstrate that PPIC is a powerful prognostic marker for HCC, and the molecular revelation of its pro-cancer mechanism might contribute to HCC treatment.

Despite its promising results, SRPS has certain limitations. Currently, SRPS is restricted to transferring known subtypes from a source cohort to target cohorts and lacks the capability to discover unified new subtypes across multiple cohorts. In future work, we aim to address this limitation by integrating SRPS with unsupervised clustering methods to enable the discovery of novel subtypes from diverse datasets.

Additionally, while we have validated the role of PPIC in this study, other subtype-significant proteins identified by SRPS have yet to be experimentally examined. Future efforts will focus on functionally validating these proteins, which may further elucidate their biological relevance and clinical utility in cancer subtyping.

## Code availability

The full code in R and Python is available via GitHub at https://github.com/PHOENIXcenter/SRPS. The code has also been submitted to BioCode at the National Genomics Data Center (NGDC), China National Center for Bioinformation (CNCB) (BioCode: BT007770), which is publicly accessible at https://ngdc.cncb.ac.cn/biocode/tool/BT007770.

## Supplementary Material

qzaf052_Supplementary_Data

## Data Availability

The processed datasets of real-world HCC and LUAD cohorts are available at https://github.com/PHOENIXcenter/SRPS.

## CRediT author statement

**Linhai Xie:** Conceptualization, Data curation, Formal analysis, Methodology, Software, Validation, Visualization, Writing – original draft. **Pei Jiang:** Investigation, Methodology, Resources, Visualization, Writing – original draft. **Cheng Chang:** Conceptualization, Formal analysis, Funding acquisition, Supervision, Writing – review & editing. All authors have read and approved the final manuscript.
