# Supplementary material for: SRPS: Survival Reinforced Transfer Learning for Multicentric Proteomic Subtyping and Biomarker Discovery
Source: Genomics Proteomics Bioinformatics. 2025 Jun 10;23(5):qzaf052. doi: 10.1093/gpbjnl/qzaf052 (PMC13005944; doi:10.1093/gpbjnl/qzaf052)
Supplement: qzaf052_Supplementary_Data [file qzaf052_supplementary_data.zip › supplementary material captions.docx]

**Supplementary materials**

**Figure S1 The visualization of 121 toy datasets used for proof of concepts**

The features in source and target cohorts are originally identical without batch effects and the survival time between two subtypes are strictly separated. The survival time of S-I samples is all longer than 30 months while the survival time of S-II samples are all shorter than 30 months. We polluted the feature with 10 different levels of random noise to simulate batch effects. Meanwhile, we also randomly swapped the survival time between two subtypes with 10 different swapping rates to decrease the correlation between subtypes and survival time. It finally resulted in 11×11=121 datasets.

**Figure S2 The visualization of simulated datasets for benchmarking**

The 3D t-SNE visualization of simulated data with different levels of batch effects (**A** and **B**) and the Kaplan-Meier curves of the synthetic prognosis of each subtype in the target cohort (**C** and **D**). Each color indicates a subtype while dots and crosses represent samples from source cohort and target cohort respectively.

**Figure S3 The distributional divergence between synthetic data and real-world proteomic data**

In this experiment, we randomly sampled data from the simulated dataset (generated using the Splatter package) and two real-world HCC datasets used in our study (Jiang et al.’s cohort and Gao et al.’s cohort). The synthetic data was preprocessed using log2​ transformation, and all datasets were further processed with zero-value imputation and Z-score normalization. For each dataset, five samples were selected. The first three subfigures depict the normalized expression/abundance distributions of the samples from each dataset, with the bold black curve representing the average distribution. The final subfigure shows the Jensen–Shannon Divergence (JSD), a symmetric distance metric between distributions, calculated between the average distributions of the datasets. The results demonstrate that the divergence between the synthetic data and Jiang et al.’s cohort is as close as the divergence between the two real-world HCC datasets, which were generated using distinct proteomic quantification methods (Label-Free and TMT-labeling).

**Figure S4 Comparing the difficulty of the prognosis modeling on the synthetic dataset and real-word datasets**

Kaplan-Meier curves show the overall survival distributions for 100 samples randomly drawn from the synthetic dataset, Jiang et al.’s HCC cohort, and Gao et al.’s HCC cohort. The simulated survival curve is comparable to Gao et al.’s cohort. Prognostic performance was assessed by training a Random Survival Forest (RSF) model on each dataset with a 7:3 train-test split. The C-index values are presented, showing similar levels of prediction difficulty between the synthetic dataset and Gao et al.’s cohort.

**Figure S5 More advanced domain adaptation techniques did not improve proteomic subtyping**

**A.** The subtyping accuracy (mean ± standard deviation) of the supervised learning method (DNN) and domain adaptation approaches, including domain adversarial neural network (DANN), conditional domain adversarial network (CDAN), the improved version of CDAN with entropy (CDAN+E) and maximum classifier discrepancy (MCD), on simulated datasets with batch effects. It is shown that more advanced approaches such as (CDAN+E) and MCD performed worse than DANN. **B.** The accuracy curves of different domain adaptation approaches on MNIST (greyscale) and MNIST-M (colorful) datasets. Domain adaptation approaches were tested with a simple encoder (two layers of CNN, solid lines) or a complex one (three layers of CNN together with batch normalization modules, dashed lines). It proved that the performance gain of (CDAN+E) or MCD largely stems from the exploitation of a powerful image encoder. Since only fully connected neural layers were applied as the encoder for processing omics data in our experiments, DANN was selected for benchmarking with domain adaptation techniques.

**Figure S6 The rank of protein significance measured by the mean** $\text{Δweight}$ **converged during the optimization process**

It was calculated based on the averaged $\text{Δweight}$ of 25 SRPS models (5 folds × 5 repeats). Here, we picked the top-10 proteins in the final models. It can be inferred from the figure that these proteins ranked randomly at the beginning of the training due to the random initialization but finally converged at high ranks. It proves that 10000 steps of optimization are enough to get a stable ranking result.

**Figure S7 Stability of the subtype significance score with varying numbers of models**

Curves depict the relationship between the rate of stable rankings in top-K ranked proteins and the number of models used to calculate the subtype significance score. Stability is measured by the consistency of rankings over the last three training epochs across 100 models trained with different random seeds. Each curve corresponds to a specific K value (10, 30, 100, 300, and 1000). For top-30 protein analysis, a minimum of 19 models ensures 95% stability in the ranking of subtype significance scores.

**Figure S8 The visualization of Jiang et al.’s cohort**

**A.** The pathway enrichment scores of the originally discovered subtypes. **B.** The Kaplan-Meier curves of overall survival and recurrence-free survival of the originally discovered subtypes.

**Figure S9 The data heterogeneity between four real-world proteomic datasets**

The proteomic profile matrix of four real-world cohorts is visualized with 2D t-SNE. Samples belonging to each cohort aggregated into separatable clusters, demonstrating unignorable inter-cohort heterogeneity.

**Figure S10 The visualization of subtyping results on Gao et al.’s cohort**

**A.** The pathway enrichment scores of the SRPS transferred subtypes. **B.** The Kaplan-Meier curves of overall survival and recurrence-free survival of the SRPS transferred subtypes.

**Figure S11 The visualization of subtyping results on Xing et al.’s cohort**

**A.** The pathway enrichment scores of the SRPS transferred subtypes. **B.** The Kaplan-Meier curves of overall survival and recurrence-free survival of the SRPS transferred subtypes.

**Figure S12 The visualization of subtyping results on Xu et al.’s cohort**

**A.** The pathway enrichment scores of the SRPS transferred subtypes. **B.** The Kaplan-Meier curves of overall survival and recurrence-free survival of the SRPS transferred subtypes.

**Figure S13 PPIC promotes cell migration and cell colony formation in HCC cell lines**

**A.** Relative mRNA expressions of *PPIC* after the indicated treatment. **B.** Resulting cell colonies (left) and the related statistical graphs (right) after the indicated treatment. **C.** Resulting staining pictures obtained from cell migration assay after the indicated treatment. Data were presented as mean ± Standard Error of the Mean (SEM). *, *P* < 0.05.

**Table S1 Datasets used in experiments**

**Table S2 Results of functional experiments** **in other cancer types from literature regarding the six under-explored HCC-related proteins discovered by SRPS**
