# Supplementary material for: SRPS: Survival Reinforced Transfer Learning for Multicentric Proteomic Subtyping and Biomarker Discovery
Source: Genomics Proteomics Bioinformatics. 2025 Jun 10;23(5):qzaf052. doi: 10.1093/gpbjnl/qzaf052 (PMC13005944; doi:10.1093/gpbjnl/qzaf052)
Supplement: qzaf052_Supplementary_Data [file qzaf052_supplementary_data.zip › Table S1.docx]

| Dataset | Data type | No. of feature used | Clinical outcome | No. of samples |
| --- | --- | --- | --- | --- |
| 121×2 toy datasets | Simulated data | 20 | Survival Time | 250 / dataset |
| 2 splatter datasets (without batch effect) | Simulated data | 1000 | Survival Time | 500 / dataset |
| 2 splatter datasets (With batch effect) | Simulated data | 1000 | Survival Time | 500 / dataset |
| Jiang et al.’s HCC cohort | Real-world proteomics data | 1097 | OS & RFS | 101 |
| Gao et al.’s HCC cohort | Real-world proteomics data | 1097 | OS & RFS | 159 |
| Xing et al.’s HCC cohort | Real-world proteomics data | 1097 | OS & RFS | 152 |
| Xu et al.’s LUAD cohort | Real-world proteomics data | 1097 | OS & RFS | 103 |

**Table S1 Datasets used in experiments**

*Note*: HCC, hepatocellular carcinoma; LUAD, lung adenocarcinoma; OS, overall survival; RFS, recurrence-free survival.
