# Supplementary material for: SRPS: Survival Reinforced Transfer Learning for Multicentric Proteomic Subtyping and Biomarker Discovery
Source: Genomics Proteomics Bioinformatics. 2025 Jun 10;23(5):qzaf052. doi: 10.1093/gpbjnl/qzaf052 (PMC13005944; doi:10.1093/gpbjnl/qzaf052)
Supplement: qzaf052_Supplementary_Data [file qzaf052_supplementary_data.zip › Table S2.docx]

| **Protein name** | **Abbrev.** | **Cancer type** | **Results of functional experiments** | **Ref.** |
| --- | --- | --- | --- | --- |
| Peptidyl-prolyl cis-trans isomerase C | PPIC | Melanoma | PPIC was identified as a promoter of melanoma progression, enhancing cell invasiveness while concurrently suppressing CD8^+^ T cell activation. | [1] |
|  |  | Gastric cancer | PPIC as a gene highly expressed in CAFs was closely associated with NCT (Neoadjuvant chemotherapy) resistance in gastric cancer. | [2] |
| Coronin-7 | CORO7 | - | - | - |
| Tubulin polymerization-promoting protein family member 3 | TPPP3 | Glioblastoma | TPPP3 promote epithelial-mesenchymal transition via Snail1 in glioblastoma. | [3] |
|  |  | Endometrial carcinoma | TPPP3 knockdown displayed evident suppression in cell proliferation, migration and invasion *in vitro* and *in vivo*. | [4] |
|  |  | Breast cancer | Silence of TPPP3 suppresses cell proliferation, invasion and migration via inactivating NF-κB/COX2 signal pathway. | [5] |
|  |  | Non-small-cell lung cancer | Knockdown of TPPP3 suppresses proliferation and induces apoptosis in non-small-cell lung cancer. | [6] |
|  |  | Non-small-cell lung cancer | TPPP3 promotes cell proliferation, invasion and tumor metastasis via STAT3/ Twist1 pathway in non-small-cell lung carcinoma. | [7] |
|  |  | Lewis lung carcinoma | Stable knockdown of TPPP3 by RNA interference in Lewis lung carcinoma cell inhibits tumor growth and metastasis. | [8] |
|  |  | Colorectal cancer | Knockdown of TPPP3 inhibits cell proliferation and invasion. | [9] |
|  |  | Nasopharyngeal carcinoma | TPPP3 overexpression inhibits cell proliferation and invasion in nasopharyngeal carcinoma. | [10] |
| Rho-related GTP-binding protein RhoG | RHOG | Salivary adenoid cystic carcinoma | RhoG overexpression promoted SACC-83 cell migration and invasion through activating Rac1. | [11] |
|  |  | Glioblastoma | The miR‑124‑3p interacted with RhoG, inhibited RhoG expression and suppressed glioblastoma cell proliferation and migration. | [12] |
|  |  | Glioblastoma | siRNA-mediated depletion of RhoG inhibits colony formation and invasion of glioblastoma cells. | [13]{Kwiatkowska, 2012 #775} |
| WASH complex subunit 3 | WASHC3 | - | - | - |
| Kinesin light chain 1 | KLC1 | Breast cancer | Loss of KLC1 inhibited cell metastasis and stem cell markers expression. | [14]{Moamer, 2019 #776} |
|  |  | Glioma | KLC1-ROS1 fusion upregulated cell proliferation, invasion, and chemoresistance when compared to wild-type ROS1. | [15]{Fujii, 2023 #777} |

**Table S2 Results of functional experiments** **in other cancer types from literature regarding the six under-explored HCC-related proteins discovered by SRPS**

**References**

[1] Zhou B, Sha S, Wang Q, Sun S, Tao J, Zhu J, et al. The prognostic implications of cuproptosis-related gene signature and the potential of PPIC as a promising biomarker in cutaneous melanoma. Pigment Cell Melanoma Res 2024;37:864–80.

[2] Yin H, Sun L, Yuan Y, Zhu Y. PPIC-labeled CAFs: key players in neoadjuvant chemotherapy resistance for gastric cancer. Transl Oncol 2024;48:102080.

[3] Xu X, Hou Y, Long N, Jiang L, Yan Z, Xu Y, et al. TPPP3 promote epithelial-mesenchymal transition via Snail1 in glioblastoma. Sci Rep 2023;13:17960.

[4] Shen A, Tong X, Li H, Chu L, Jin X, Ma H, et al. TPPP3 inhibits the proliferation, invasion and migration of endometrial carcinoma targeted with miR-1827. Clin Exp Pharmacol Physiol 2021;48:890–901.

[5] Ren Q, Hou Y, Li X, Fan X. Silence of TPPP3 suppresses cell proliferation, invasion and migration via inactivating NF-κB/COX2 signal pathway in breast cancer cell. Cell Biochem Funct 2020;38:773–81.

[6] Li Y, Xu Y, Ye K, Wu N, Li J, Liu N, et al. Knockdown of tubulin polymerization promoting protein family member 3 suppresses proliferation and induces apoptosis in non-small-cell lung cancer. J Cancer 2016;7:1189–96.

[7] Li Y, Bai M, Xu Y, Zhao W, Liu N, Yu J. TPPP3 promotes cell proliferation, invasion and tumor metastasis via STAT3/ Twist1 pathway in non-small-cell lung carcinoma. Cell Physiol Biochem 2018;50:2004–16.

[8] Zhou W, Li J, Wang X, Hu R. Stable knockdown of TPPP3 by RNA interference in Lewis lung carcinoma cell inhibits tumor growth and metastasis. Mol Cell Biochem 2010;343:231–8.

[9] Ye K, Li Y, Zhao W, Wu N, Liu N, Li R, et al. Knockdown of tubulin polymerization promoting protein family member 3 inhibits cell proliferation and invasion in human colorectal cancer. J Cancer 2017;8:1750–8.

[10] Su Q, Yang Z, Guo X, Mo W, Li X. Tubulin polymerization promoting protein family member 3 (TPPP3) overexpression inhibits cell proliferation and invasion in nasopharyngeal carcinoma. Bioengineered 2021;12:8485–95.

[11] Xu ZD, Hao T, Gan YH. RhoG/Rac1 signaling pathway involved in migration and invasion of salivary adenoid cystic carcinoma cells. Oral Dis 2020;26:302–12.

[12] Cai S, Shi CJ, Lu JX, Wang YP, Yuan T, Wang XP. miR‑124‑3p inhibits the viability and motility of glioblastoma multiforme by targeting RhoG. Int J Mol Med 2021;47:1–13.

[13] Kwiatkowska A, Didier S, Fortin S, Chuang Y, White T, Berens ME, et al. The small GTPase RhoG mediates glioblastoma cell invasion. Mol Cancer 2012;11:65.

[14] Moamer A, Hachim IY, Binothman N, Wang N, Lebrun J-J, Ali S. A role for kinesin-1 subunits KIF5B/KLC1 in regulating epithelial mesenchymal plasticity in breast tumorigenesis. EBioMedicine 2019;45:92–107.

[15] Fujii T, Nakano Y, Hagita D, Onishi N, Endo A, Nakagawa M, et al. KLC1-ROS1 fusion exerts oncogenic properties of glioma cells via specific activation of JAK-STAT pathway. Cancers 2023;11.
