# Supplementary figures and images for: SRPS: Survival Reinforced Transfer Learning for Multicentric Proteomic Subtyping and Biomarker Discovery
Source: Genomics Proteomics Bioinformatics. 2025 Jun 10;23(5):qzaf052. doi: 10.1093/gpbjnl/qzaf052 (PMC13005944; doi:10.1093/gpbjnl/qzaf052)

Source cohort

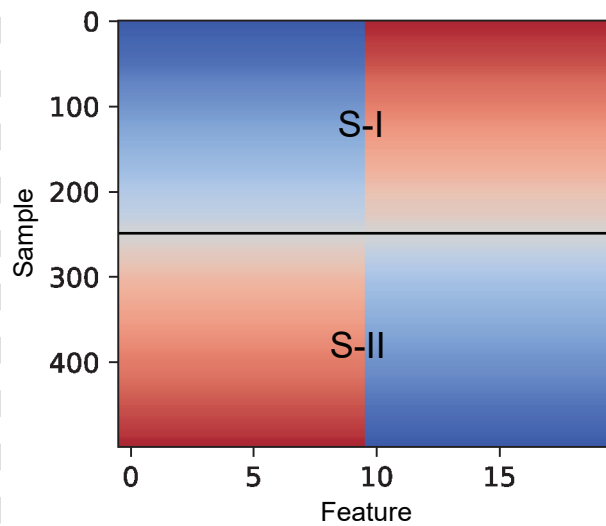

⋮

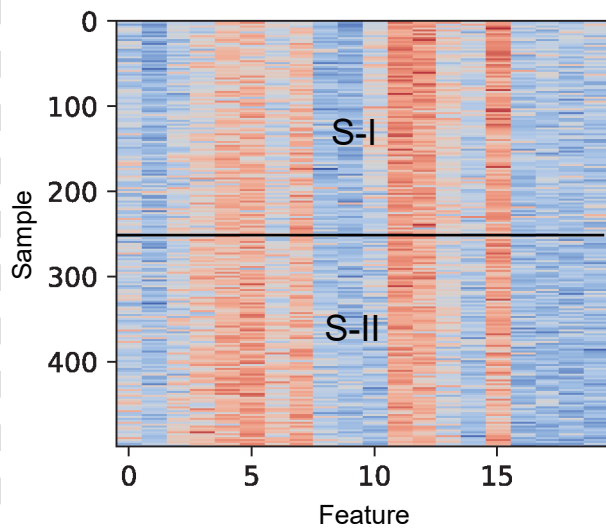

Target cohort

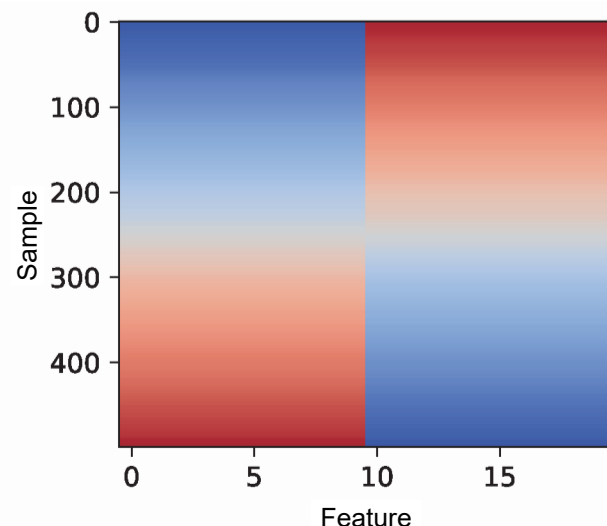

⋮

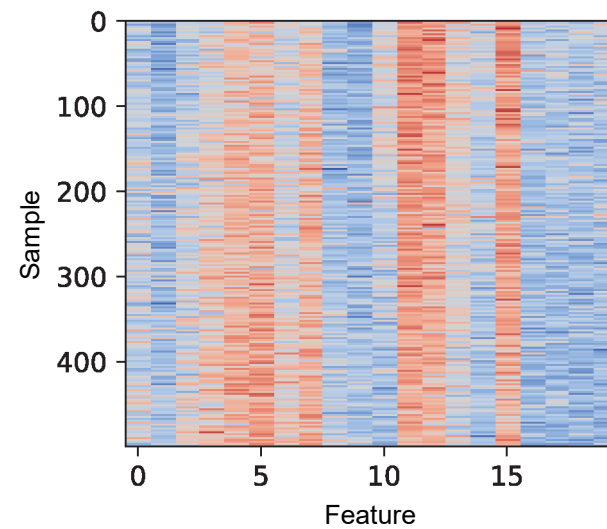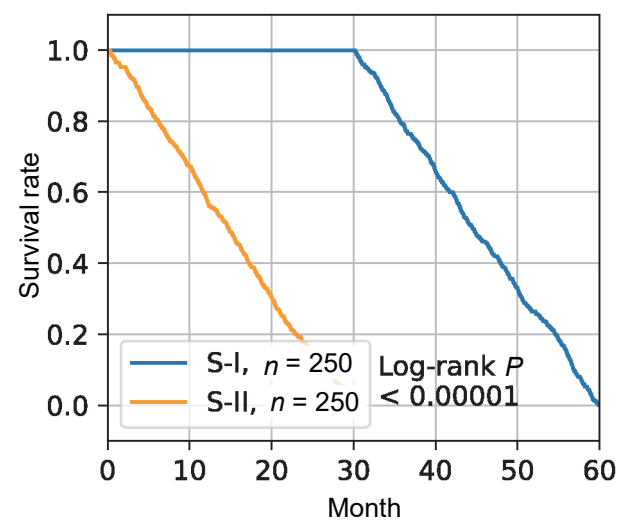

⋮

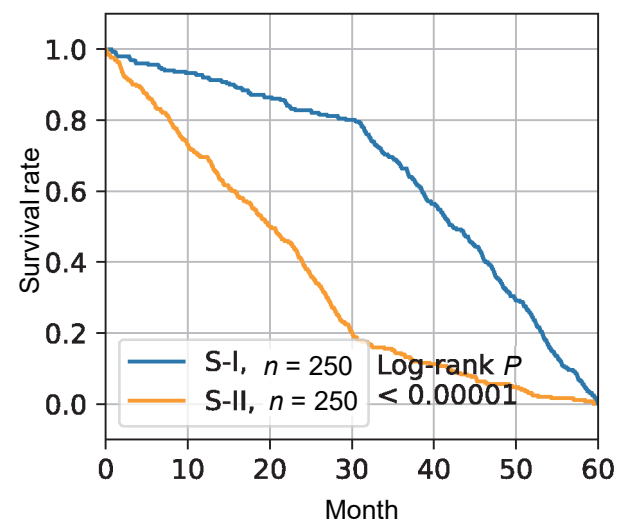

Supplement: qzaf052_Supplementary_Data [file qzaf052_supplementary_data.zip › Figure S1.pdf]

Gao et al.'s cohort

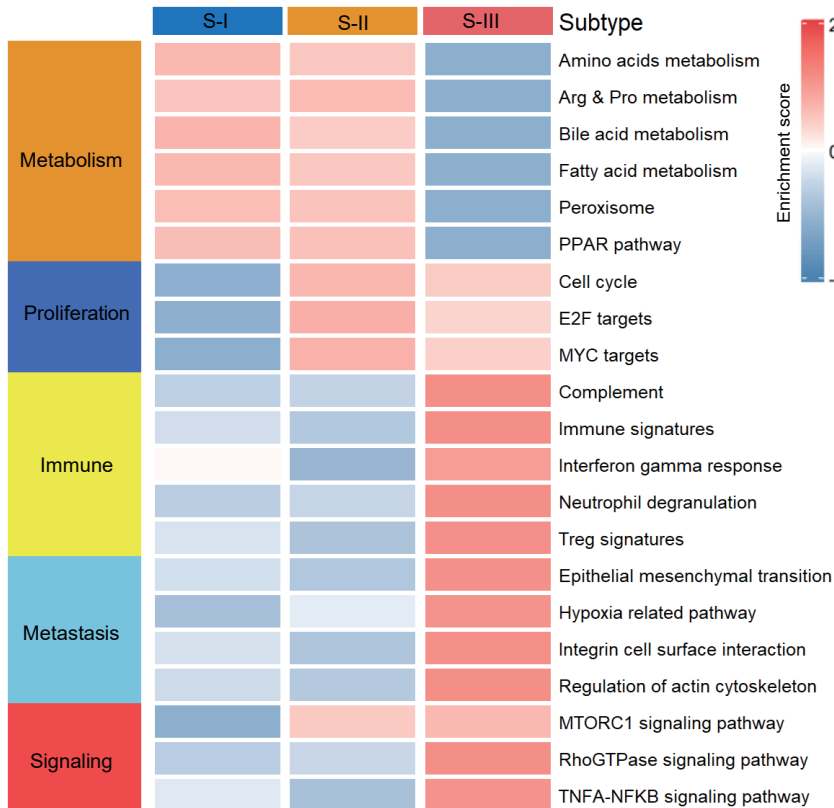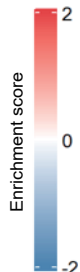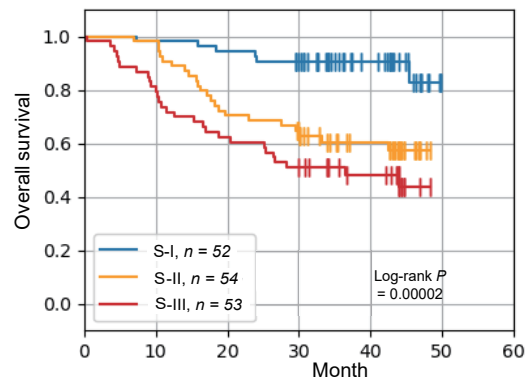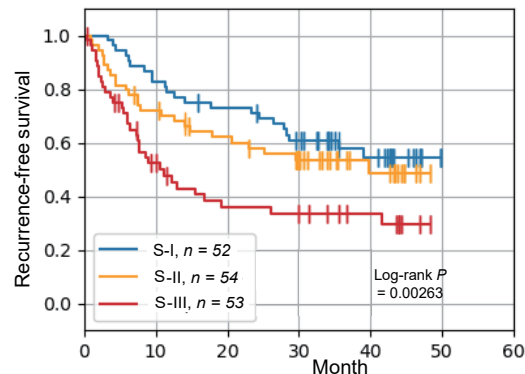

Supplement: qzaf052_Supplementary_Data [file qzaf052_supplementary_data.zip › Figure S10.pdf]

Xing et al.'s cohort

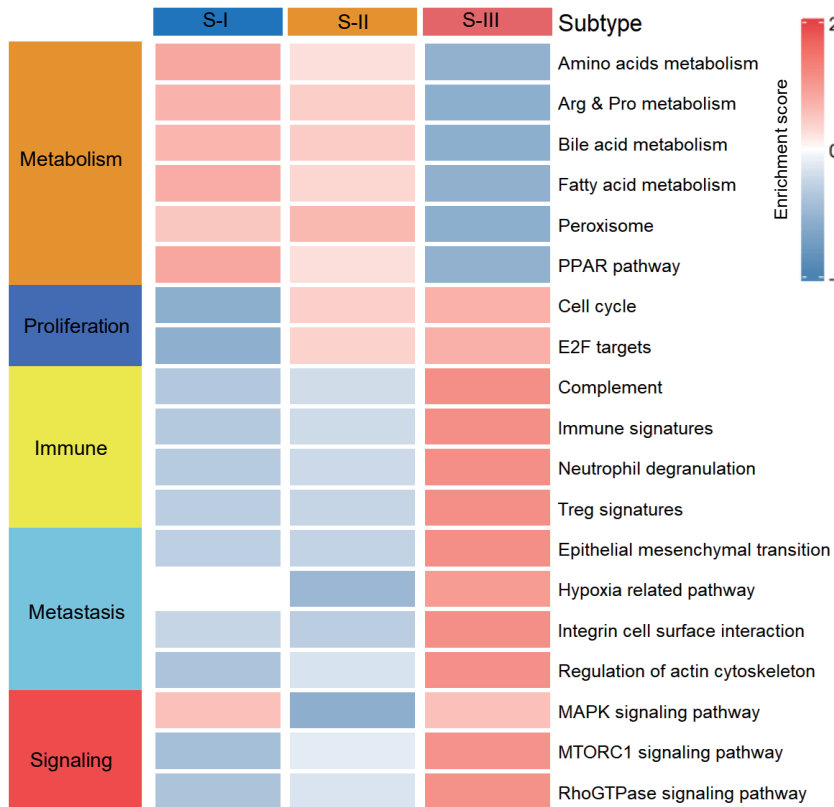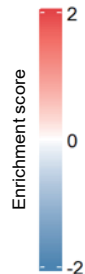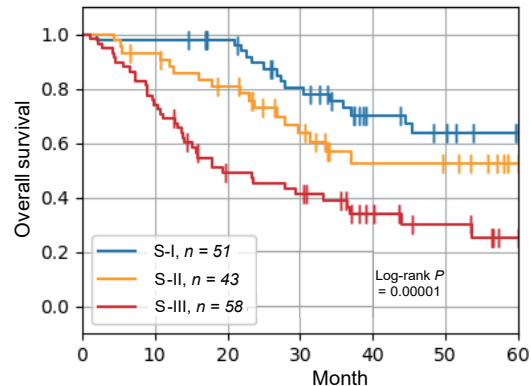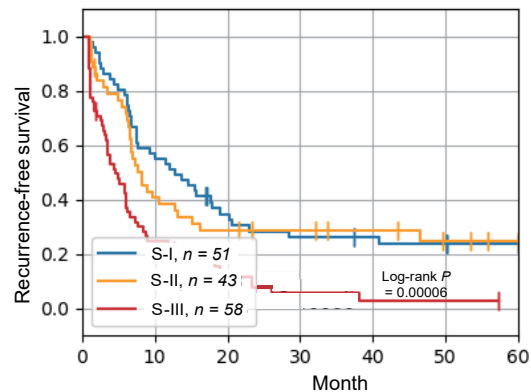

Supplement: qzaf052_Supplementary_Data [file qzaf052_supplementary_data.zip › Figure S11.pdf]

Xing et al.'s cohort

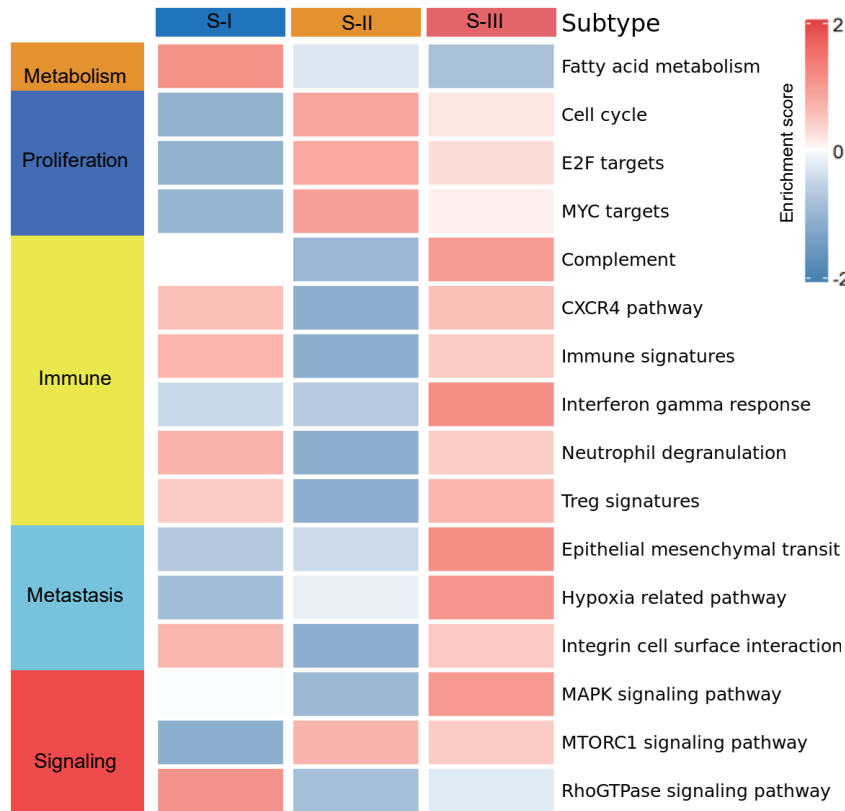

Enrichment score

2

0

-2

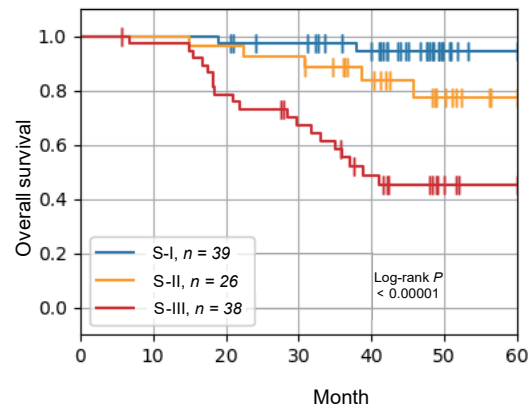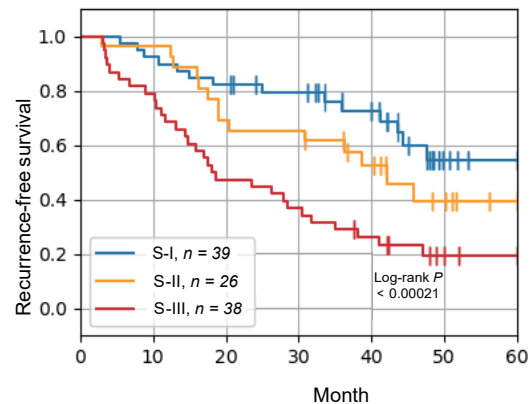

Supplement: qzaf052_Supplementary_Data [file qzaf052_supplementary_data.zip › Figure S12.pdf]

**A**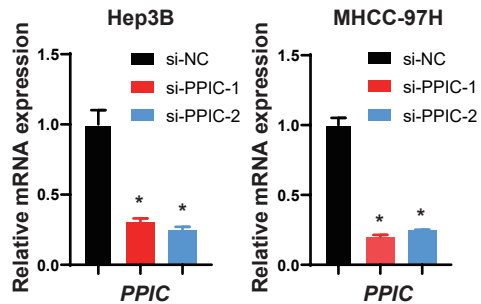**B**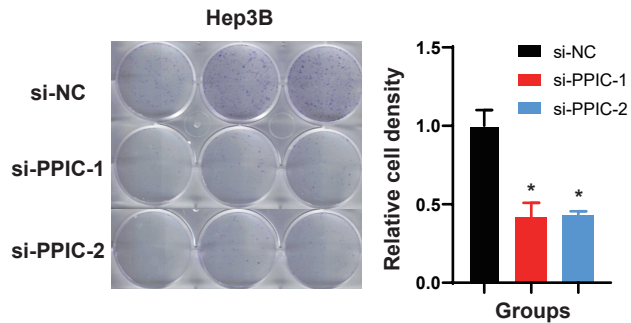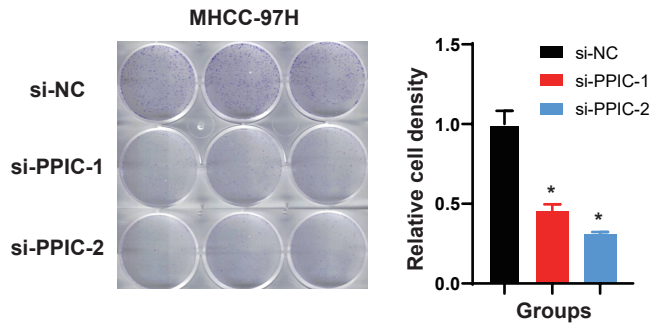**C**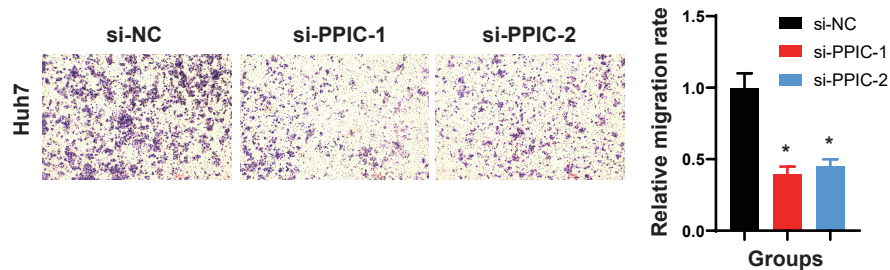

Supplement: qzaf052_Supplementary_Data [file qzaf052_supplementary_data.zip › Figure S13.pdf]

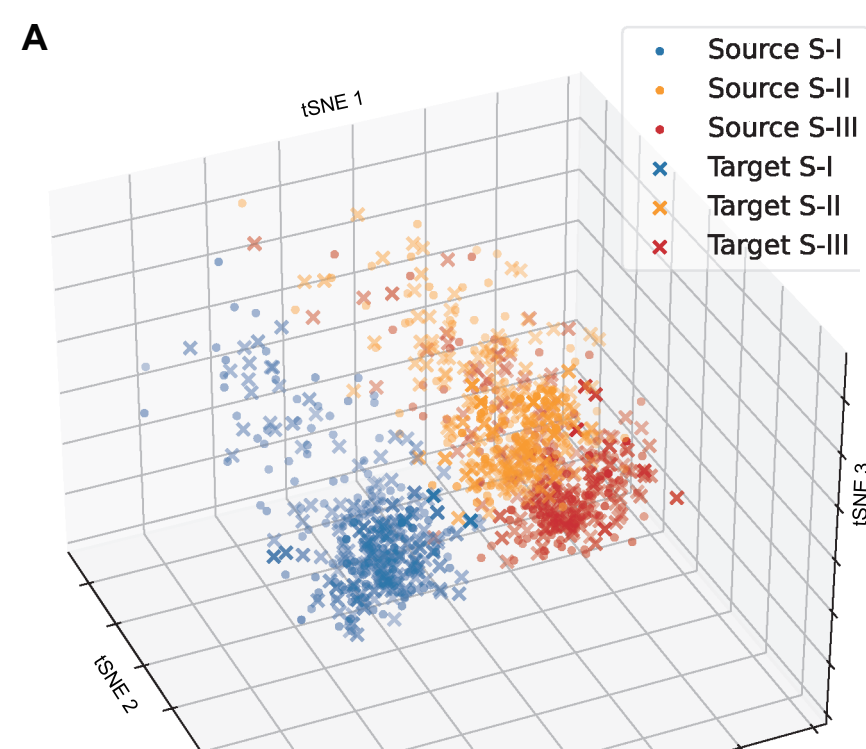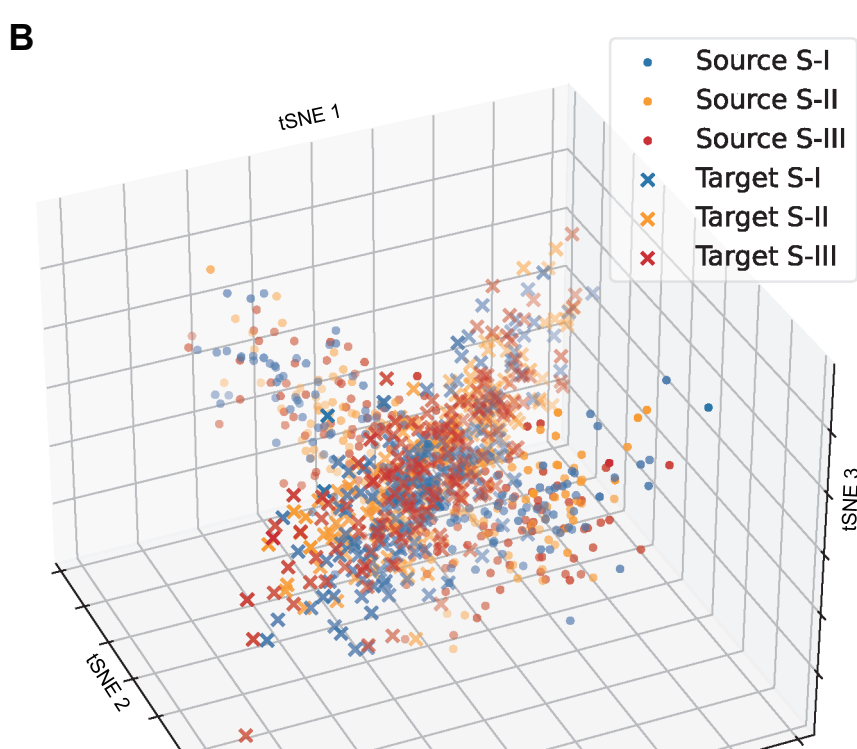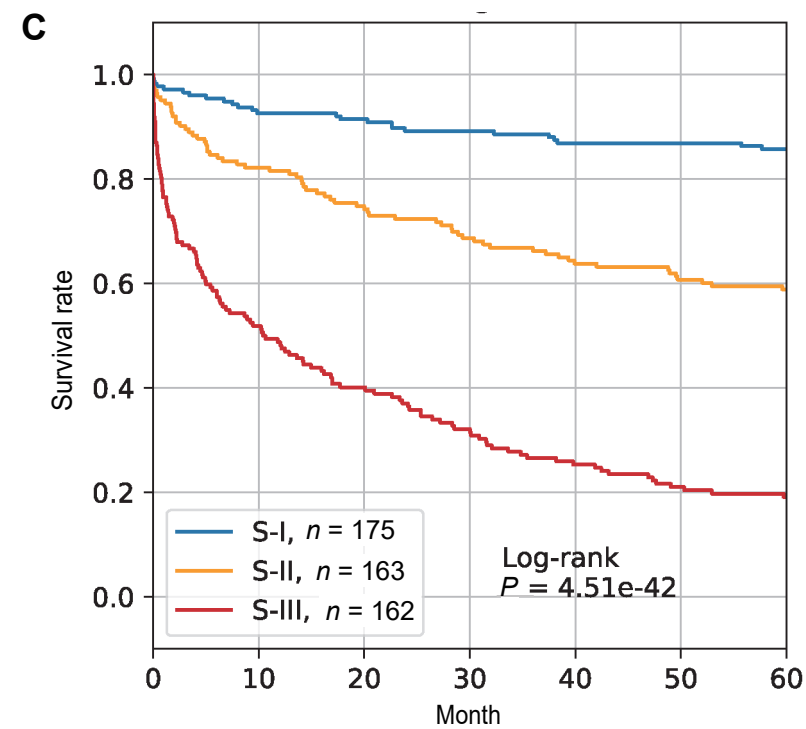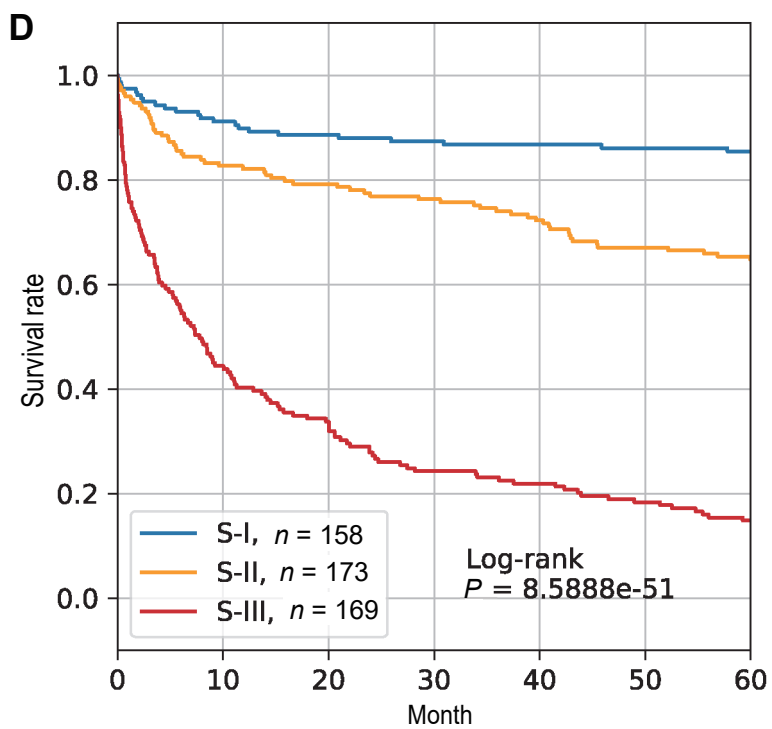

Supplement: qzaf052_Supplementary_Data [file qzaf052_supplementary_data.zip › Figure S2.pdf]

Synthetic data

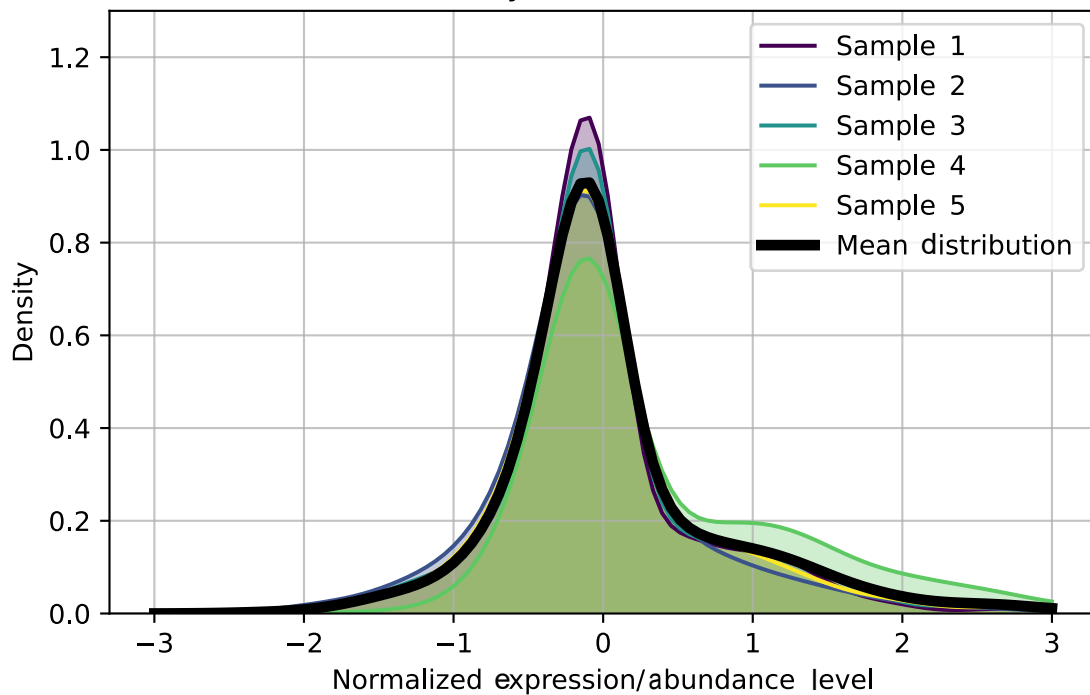

Jiang et al.'s HCC data

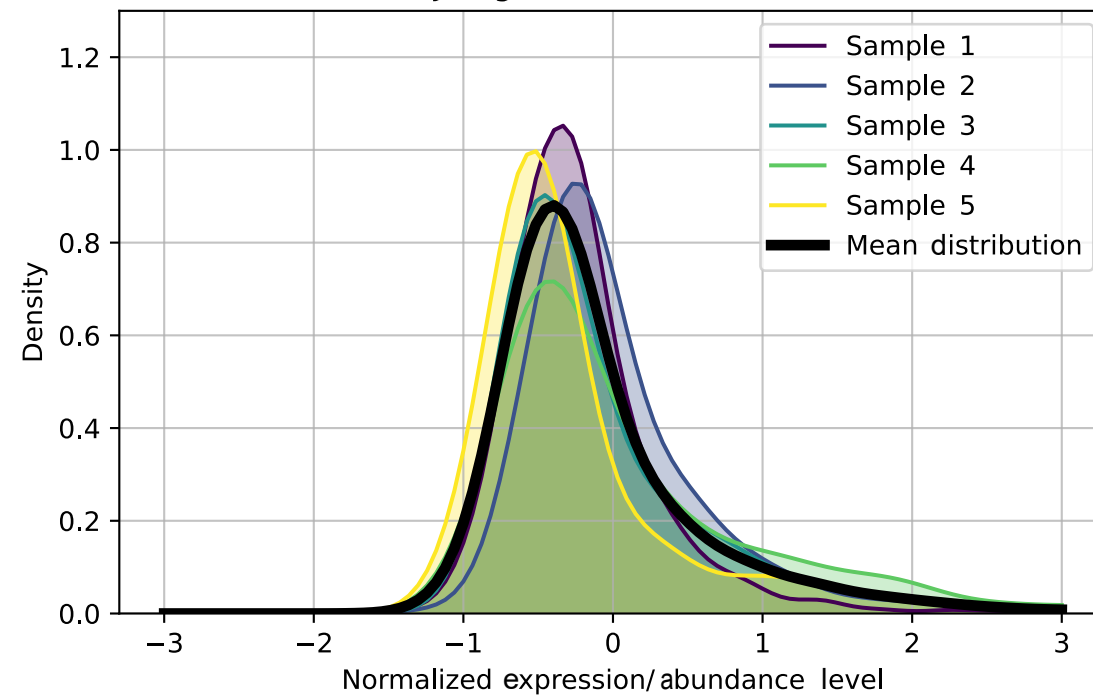

Gao et al.'s HCC data

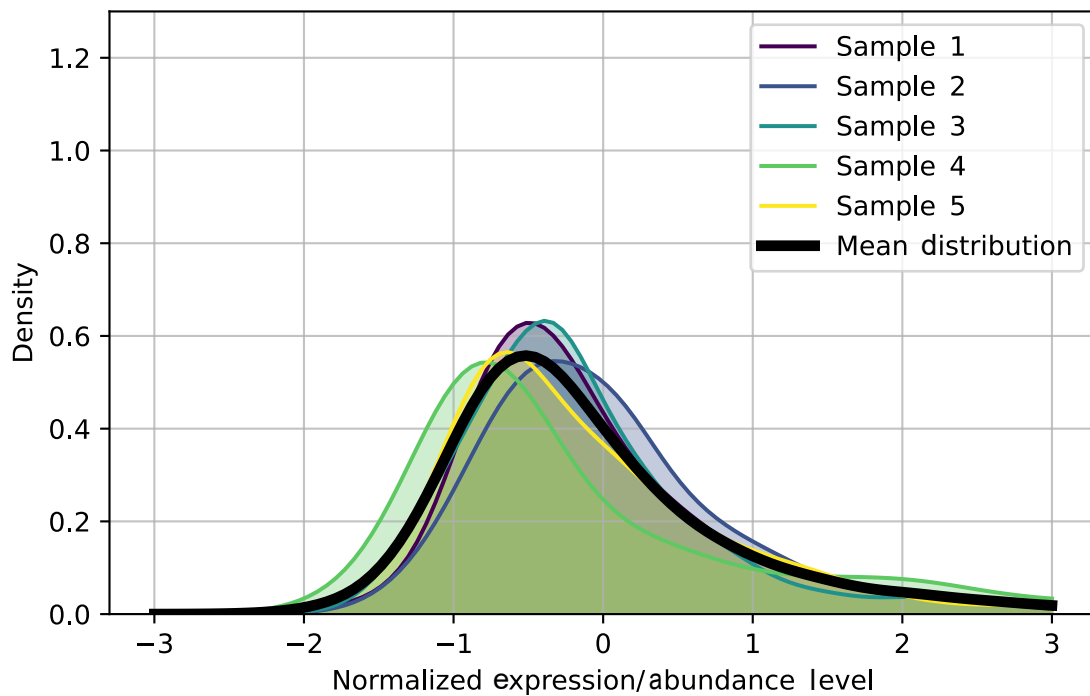

Comparison of mean distribution curves

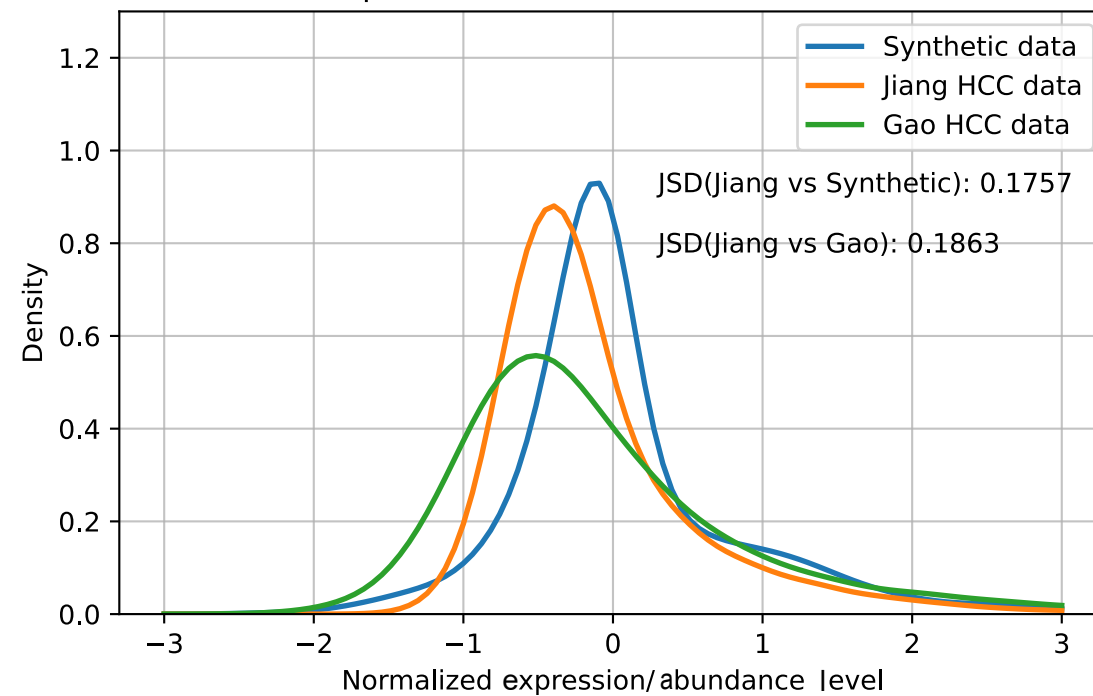

Supplement: qzaf052_Supplementary_Data [file qzaf052_supplementary_data.zip › Figure S3.pdf]

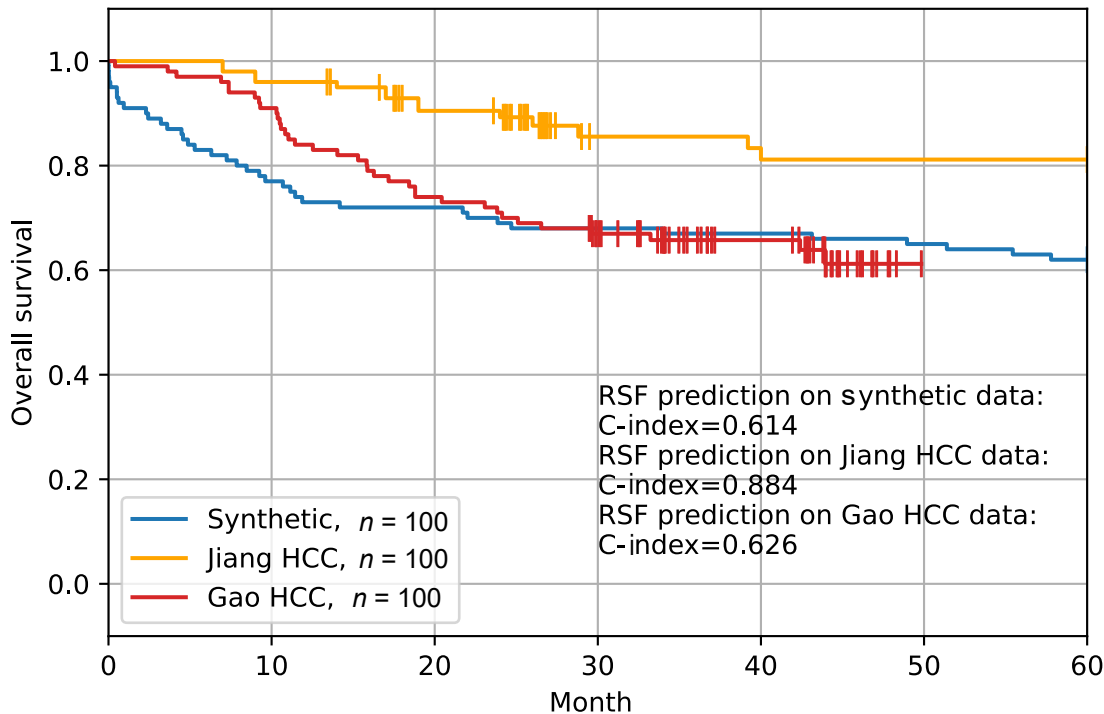

Supplement: qzaf052_Supplementary_Data [file qzaf052_supplementary_data.zip › Figure S4.pdf]

**A**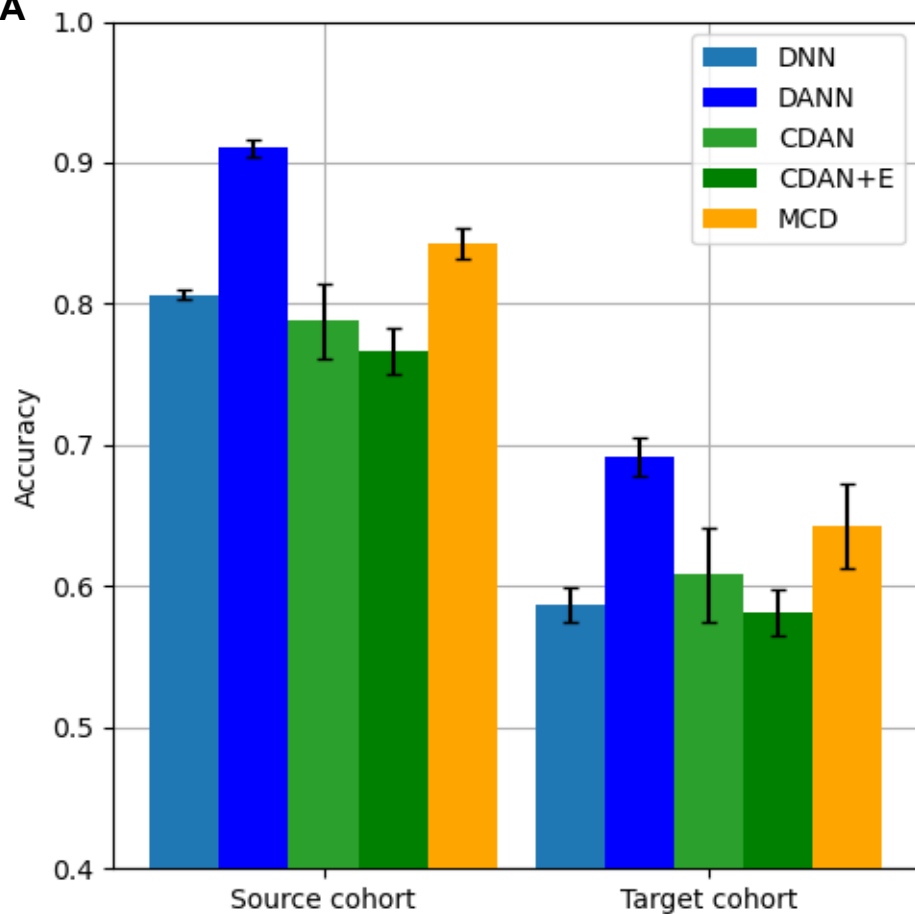**B**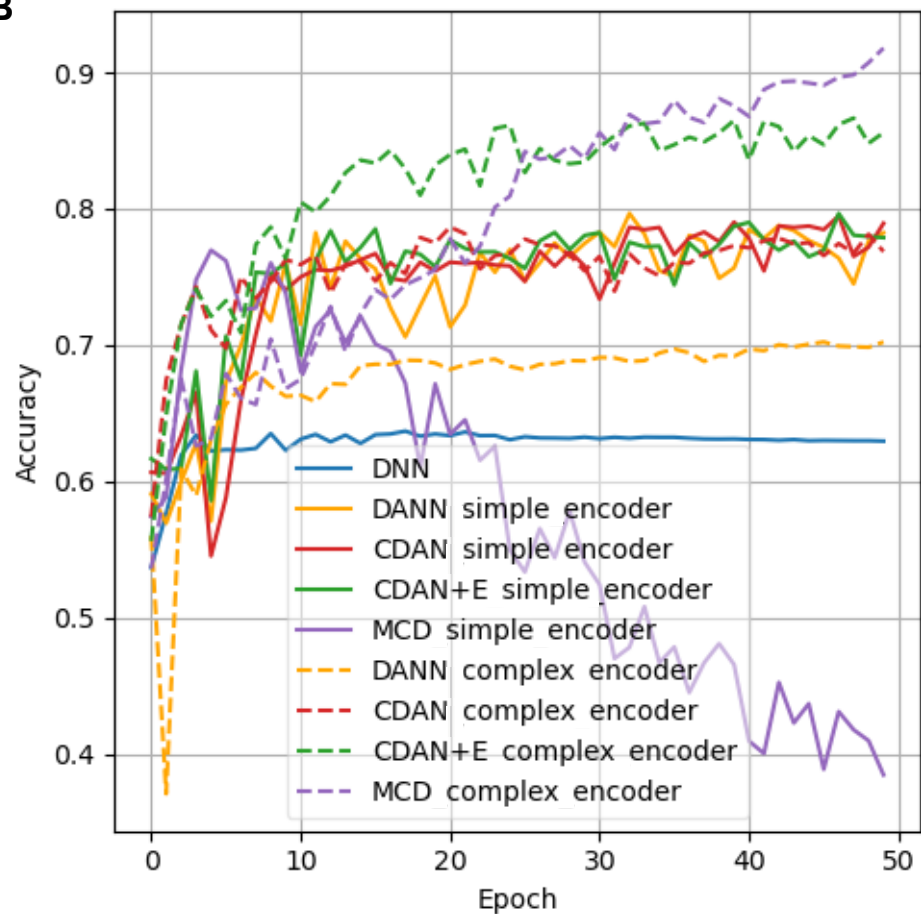

Supplement: qzaf052_Supplementary_Data [file qzaf052_supplementary_data.zip › Figure S5.pdf]

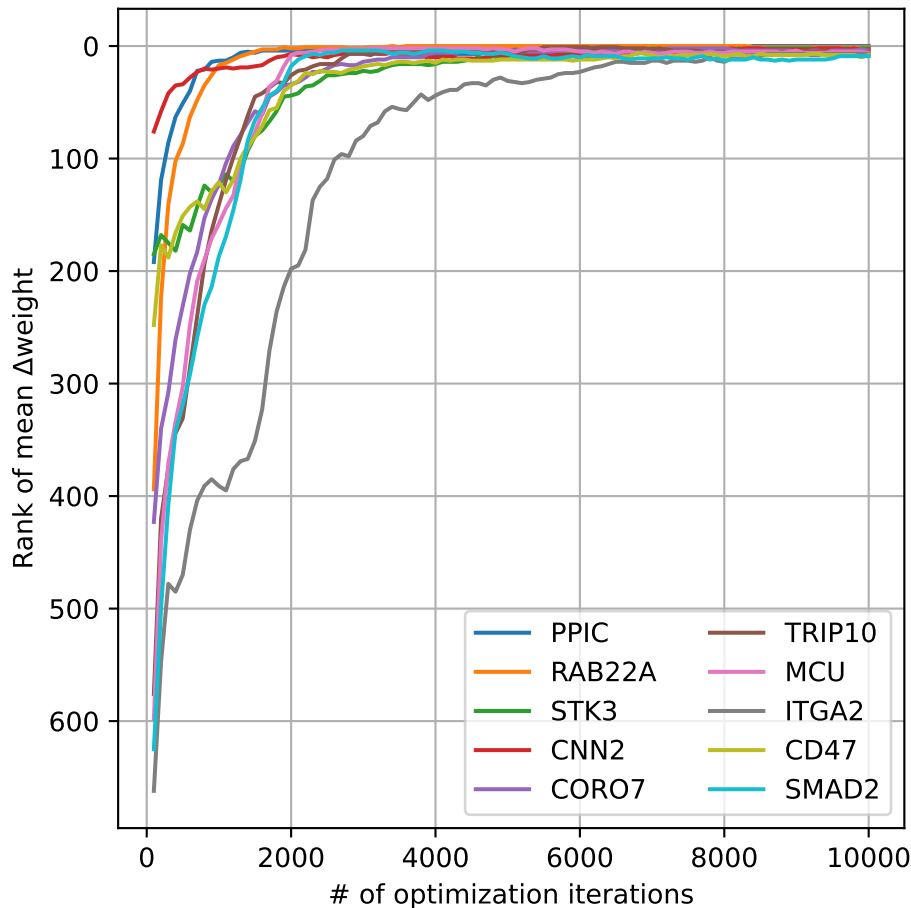

Supplement: qzaf052_Supplementary_Data [file qzaf052_supplementary_data.zip › Figure S6.pdf]

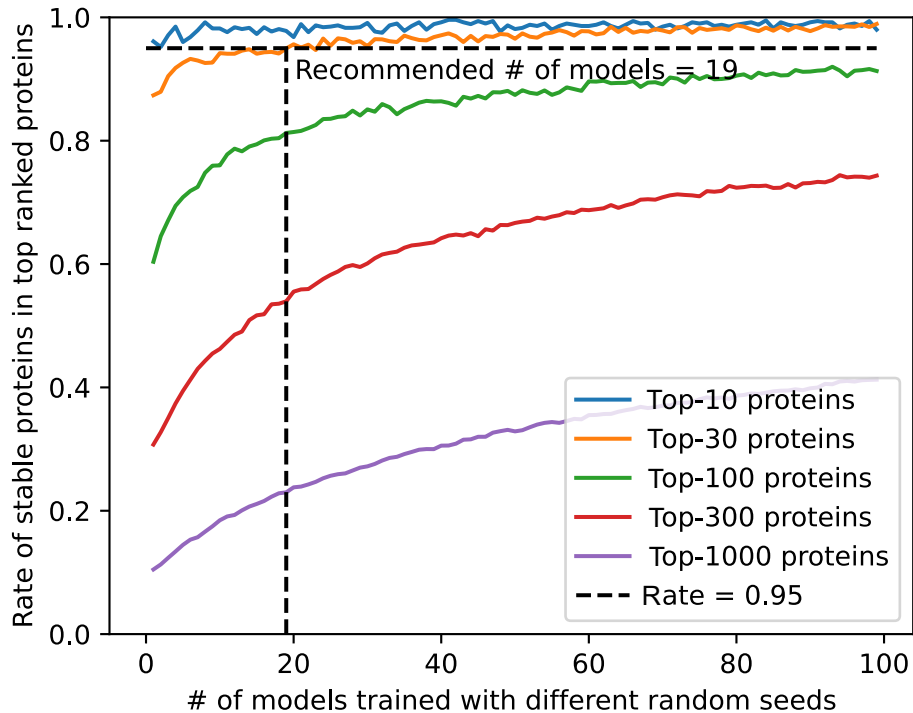

Supplement: qzaf052_Supplementary_Data [file qzaf052_supplementary_data.zip › Figure S7.pdf]

Jiang et al.'s cohort

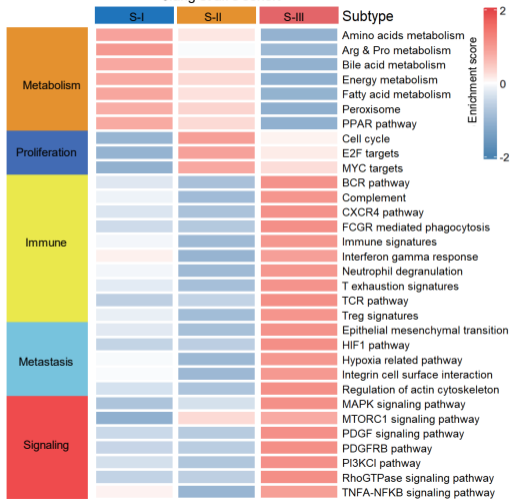

Enrichment score

2

0

-2

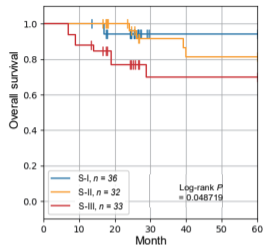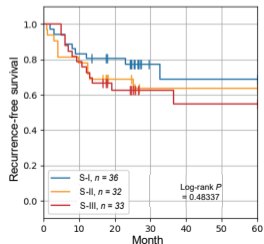

Supplement: qzaf052_Supplementary_Data [file qzaf052_supplementary_data.zip › Figure S8.pdf]

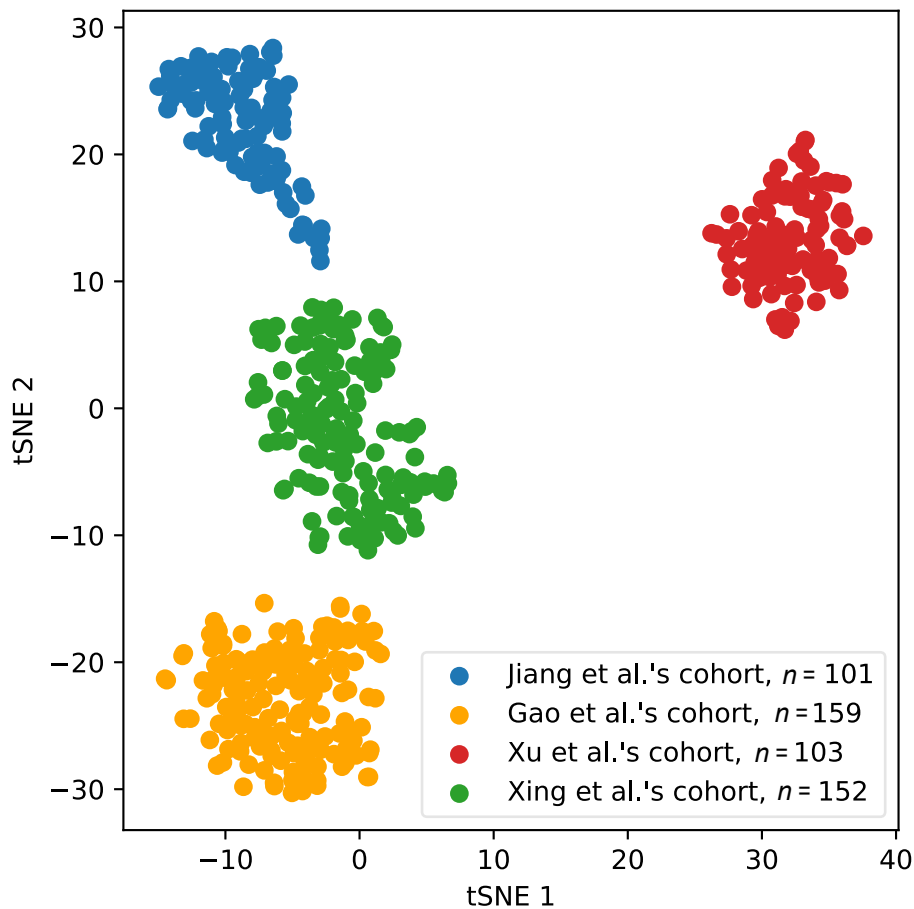

Supplement: qzaf052_Supplementary_Data [file qzaf052_supplementary_data.zip › Figure S9.pdf]
